# A Phenogenetic Axis that Modulates Clinical Manifestation and Predicts Treatment Outcome in Primary Myeloid Neoplasms

**DOI:** 10.1158/2767-9764.CRC-21-0194

**Published:** 2022-04-26

**Authors:** Qiujin Shen, Yahui Feng, Xiaowen Gong, Yujiao Jia, Qingyan Gao, Xiaokang Jiao, Saibing Qi, Xueou Liu, Hui Wei, Bingqing Huang, Ningning Zhao, Xiaoqiang Song, Yueshen Ma, Shihao Liang, Donglei Zhang, Li Qin, Ying Wang, Shiqiang Qu, Yao Zou, Yumei Chen, Ye Guo, Shuhua Yi, Gang An, Zengtao Jiao, Song Zhang, Linfeng Li, Jun Yan, Huijun Wang, Zhen Song, Yingchang Mi, Lugui Qiu, Xiaofan Zhu, Jianxiang Wang, Zhijian Xiao, Junren Chen

**Affiliations:** 1 State Key Laboratory of Experimental Hematology, National Clinical Research Center for Blood Diseases, Haihe Laboratory of Cell Ecosystem, Institute of Hematology & Blood Diseases Hospital, Chinese Academy of Medical Sciences & Peking Union Medical College, Tianjin, China.; 2 Yidu Cloud Technology Inc., Beijing, China.

## Abstract

Although the concept of “myeloid neoplasm continuum” has long been proposed, few comparative genomics studies directly tested this hypothesis. Here we report a multi-modal data analysis of 730 consecutive newly diagnosed patients with primary myeloid neoplasm, along with 462 lymphoid neoplasm cases serving as the outgroup. Our study identified a “Pan-Myeloid Axis” along which patients, genes, and phenotypic features were all aligned in sequential order. Utilizing relational information of gene mutations along the Pan-Myeloid Axis improved prognostic accuracy for complete remission and overall survival in adult patients of *de novo* acute myeloid leukemia and for complete remission in adult patients of myelodysplastic syndromes with excess blasts. We submit that better understanding of the myeloid neoplasm continuum might shed light on how treatment should be tailored to individual diseases.

**Significance:**

The current criteria for disease diagnosis treat myeloid neoplasms as a group of distinct, separate diseases. This work provides genomics evidence for a “myeloid neoplasm continuum” and suggests that boundaries between myeloid neoplastic diseases are much more blurred than previously thought.

## Introduction

Myeloid neoplasms comprise a compendium of rare diseases that ranges from neoplasms that are more common [acute myeloid leukemia (AML) and chronic myeloid leukemia (CML)] to neoplasms that are much rarer [myelodysplastic syndromes (MDS), myeloproliferative neoplasms (MPN), and MDS/MPN overlap syndromes (MDS/MPN)]. It has been proposed that myeloid neoplasms in reality form a continuum (1–5). There is as yet, however, little pan-genomics evidence for this hypothesized “myeloid neoplasm continuum”. We argue that such line of investigation is important for further advancing therapeutics for myeloid neoplasms.

Genomic studies of myeloid neoplasms in the past often examined one or two diseases at a time, using varying panels of interrogated genes. For instance, previous influential studies of mutational spectra in AML could cover anywhere between five and 111 genes (6–16). Some of these analyses focused on *de novo* AML cases (6–8, 11, 12, 16), others focused on secondary cases (13), and yet others were more accommodating in terms of patient cohort selection (9, 14). Treatment regimens also varied substantially from study to study: while anthracycline and cytarabine formed the backbone of induction therapy in most AML studies, other medicines such as colony-stimulating factors, etoposide, and all-trans retinoic acid also played prominent roles in the induction therapy in some of the published studies (6, 9, 14, 16). Variability in research design makes it difficult to summarize across studies. Nonetheless, despite much heterogeneity across studies, most analyses agreed that CEBPA and NPM1 mutations were associated with favorable prognosis and that RUNX1, ASXL1, and TP53 mutations as well as FLT3-ITD (internal tandem repeats) were associated with adverse prognosis; common observations as such have been incorporated into the 2017 European LeukemiaNet (ELN) scheme for risk stratification by genetics (17). Note, however, that–despite supporting evidence from various reports (9, 11–14)–the 2017 ELN risk stratification did not consider the mutational status of BCOR, DNMT3A, EZH2, IDH2, SF3B1, SRSF2, STAG2, U2AF1, WT1, or ZRSR2; nor did the 2017 ELN risk stratification consider partial tandem duplication of the KMT2A gene. The Revised Acute Myeloid Leukemia Composite Model (AML-CM) proposed by Sorror and colleagues in 2019 followed the 2017 ELN scheme faithfully when counting cytogenetic/molecular aberrations and did not incorporate additional gene mutations (18).

Some AML cases are the results of transformation from previously diagnosed MDS, MPN, or MDS/MPN, and such patients with “secondary AML” have poorer prognosis than *de novo* AML (19) and often carry so-called “secondary-type” gene mutations in SRSF2, SF3B1, U2AF1, ZRSR2, ASXL1, EZH2, BCOR, or STAG2 (13). In addition, some of the *de novo* AML cases carry multi-lineage dysplasia that are suggestive of MDS; such “AML with myelodysplasia-related changes” (AML-MRC) cases have lower probability of complete remission and shorter survival (20, 21), and furthermore their mutational spectra are statistically indistinguishable from “MDS with excess blasts” (MDS-EB) (22). It is also possible for *de novo* AML cases without complex karyotypes or myelodysplasia-related changes to carry “secondary-type” gene mutations, and it has been reported that such cases have poorer prognosis (13, 23). Although there is significant correlation between AML-MRC and “secondary-type” gene mutations, a high percentage of AML cases that carry “secondary-type” mutations are neither secondary AML (13) nor AML-MRC (23). In other words, either some of the patients with “*de novo*” AML are transiting through a prodrome that does not meet the current World Health Organization (WHO) criteria for MDS and AML-MRC, or they may be true *de novo* cases that represent hitherto unrecognized biological overlap between *de novo* AML and MDS; better understanding of clonal hematopoiesis of indeterminate potential (CHIP) might shed light on this issue in the future (24, 25).

The blurred distinction between AML and MDS has received ever more attention nowadays. Traditionally, MDS cases (even high-grade MDS) were less likely to be treated with AML-type induction therapy (2, 26), so it was often difficult to conduct “apple-to-apple” comparison of the clinical courses of AML and MDS. Nonetheless, a Seattle group has been treating patients with MDS-EB2 with AML-type therapy in some of their locally initiated studies (27). Recent analysis of the Seattle dataset showed that most of the prognostic distinctions between AML and MDS-EB2 disappeared when covariates such as age, performance status, 2017 ELN cytogenetic/molecular risk class, secondary-ness, induction therapy intensity, and allogeneic hematopoietic stem cell transplantation were taken into consideration (5). In other words, the 20% blast percentage threshold that is dictated by the current WHO guideline to distinguish between the two diagnoses might not be ideal, and some have even argued that reclassification of diseases along the AML-MDS continuum based on genetic abnormalities would be more clinically relevant than the current standard practice (5, 13). Interestingly, one small-scale study lately reported that patients with NPM1-mutated MDS and MDS/MPN might be better treated with AML-type than MDS-type therapy (28). Recent studies that investigated fine-grained cytogenetic/molecular subtypes of MDS (29, 30), MPN (31), and MDS/MPN (32) are important first steps towards precision medicine along the myeloid neoplasm continuum.

Phenotypic features have always been the bed rock for risk stratification in myeloid neoplasms, as exemplified by the AML-CM algorithm (18, 33) and the Revised International Prognostic Scoring System (IPSS-R) for MDS (34). Incorporation of cytogenetic information and gene mutations has been shown to further improve prognostic accuracy (14, 30, 31, 35), and integration of multimodal data may enable machine learning-driven personalized prescriptions routinely someday (36). Clearly, if we were to rethink the myeloid neoplasm continuum, we would also need to characterize how genetic aberrations correlate with phenotypic features throughout the continuum, i.e., across diseases. Numerous previous single-disease studies have reported correlations between gene mutations and clinical profiles. In AML, for instance, mutations in CEBPA, CSF3R, FLT3, GATA2, IDH, KIT, NPM1, and WT1 have been shown to be associated with high white blood cell count or high blast count (6, 37–43). In MDS, previous studies have reported that SF3B1 and DNMT3A mutations are associated with high platelet count (44, 45), that GATA2 mutations are associated with monocytosis (46), and that mutations in RUNX1, TP53, and NRAS are associated with severe thrombocytopenia and higher blast count (47). It has also been reported that cell morphology profiles and gene mutation patterns are correlated with each other in MDS, MDS/MPN, and secondary AML (48).

In this study, we sought to understand the distribution of genetic aberrations across primary myeloid neoplasms as well as how they correlate with clinical manifestations. The patient cohort analyzed in this study is hereafter referred to as “THAMP”’ (Tianjin Hematologic Atlas of Myeloid neoplasm Phenogenetics). THAMP included 730 consecutive newly-diagnosed cases of primary myeloid neoplasms treated at the Institute of Hematology, Chinese Academy of Medical Sciences (IHCAMS; Tianjin, China) [covering AML (*n* = 388), CML (*n* = 27), MDS (*n* = 209), MDS/MPN (*n* = 36), and MPN (*n* = 70); Supplementary Table S1], along with 462 consecutive newly-diagnosed patients of lymphoid neoplasms serving as the outgroup [covering acute lymphoblastic leukemia (ALL; *n* = 321), chronic lymphocytic leukemia (CLL; *n* = 52), and multiple myeloma (MM; *n* = 89)] (Supplementary Table S2). The outgroup was included in our analysis so as to distinguish between myeloid neoplasm-specific and lymphoid neoplasm-specific genetic aberrations. All the patients in THAMP were karyotyped and sequenced at a panel of 171 genes (“THAMP-Illumina-171”; Supplementary Table S3) before receiving therapy. We integrated the multi-modal data from the patients of primary myeloid neoplasms that encompassed gene mutations, karyotypic abnormalities, and peripheral blood and bone marrow parameters to identify higher-order patterns that had clinical implications in target genes and treatment outcome.

## Materials and Methods

### Informed Consents and Ethics Approvals

This work was entirely retrospective. At admission to the IHCAMS, each of the patients included in this study signed an informed consent form for peripheral blood sample usage and another informed consent form for bone marrow sample usage; in both of the signed forms, the patient explicitly authorized the physicians at the IHCAMS to “utilize the test samples for reasonable medical usage (including medical research)”. If the primary care oncologist of a patient had not ordered a DNA sequencing test for the patient, then this patient was eliminated from this study; that is, there was no effort made during this study to collect any additional genotypic data beyond what had been deemed necessary for routine clinical care.

Before initiation of this retrospective study, the IHCAMS Ethics Committee reviewed a research proposal for the study and issued an approval letter on 16 March 2021 [reference no. (ref. no.) NSFC2021065-EC-1]. Before manuscript drafting, the IHCAMS Ethics Committee reviewed again this study's adherence to the Declaration of Helsinki and personal data protection and issued a second approval letter on November 17, 2021 (ref. no. QTJC2021003-EC-1).

### THAMP Study Cohort

Except for myeloid neoplasm cases with history of prior chemotherapy, radiotherapy, or another hematologic disease (e.g., AML following a preceding MDS), all the consecutive patients who were newly diagnosed of AML, MDS, MDS/MPN, MPN, CML, MM, CLL, or ALL between February 20, 2020 and March 24, 2021 at the IHCAMS and whose disease samples were sequenced at a panel of 171 genes (“THAMP-Illumina-171”) before initiation of therapy were all included in the THAMP study cohort.

CLL and MM were diagnosed according to the guidelines of the International Workshop on Chronic Lymphocytic Leukemia (49) and the International Myeloma Working Group (50), respectively. The other diseases were diagnosed according to the 2016 revision to the WHO classification of myeloid neoplasms and acute leukemia (21). One neonatal patient of acute megakaryoblastic leukemia (AMeL) was diagnosed based on high megakaryocytic lineage cell counts in peripheral blood, even though circulating blast count was low (possible for AMeL; ref. 51); bone marrow aspiration was difficult to perform, and bone marrow biopsy was not conducted due to the patient's very young age. Two patients had 20% to 30% blast counts based on hematoxylin and eosin staining, but immunohistochemical staining gave values that were less than 20% (both were diagnosed as MDS-EB). AML-M3 (*n* = 39) was diagnosed by the presence of t(15;17)(q24;q21) or PML-RARA fusion gene (isoform L, V, or S); 84.6% (33/39) had t(15;17)(q24;q21), 100% (39/39) had PML-RARA, and 100% (39/39) had more than 20% promyelocyte percentage in either peripheral blood or bone marrow aspiration smear analysis.

Pretreatment clinical information of each patient was extracted from the electronic medical records at the IHCAMS, covering karyotypes, complete blood counts, and peripheral blood/bone marrow aspiration smear analysis reports. The phenotypic data that were collected at times nearest to DNA sequencing were included in THAMP. [For six of the patients with AML and five of the patients with MDS-EB, the blast count data included in THAMP did not pass the 2016 WHO diagnostic criteria; they were given definite diagnosis based on data collected at separate sampling sites (e.g., sternum instead of ilium) and/or separate times (usually within 1–2 days apart).] Missing values were filled in with mean values across all the patients.

Most of the t(12;21)(p13;q22) cases were cryptic and failed to be detected by conventional karyotypical analysis (52). Presence/absence of t(12;21)(p13;q22) was ascertained by routine PCR screening of TEL-AML1 fusion gene in the patients with ALL.

Risk scores of the patients with MDS and AML were calculated according to IPSS-R (34) and 2017 ELN (17), respectively.

Minimal residual diseases (MRD) of the patients with AML after chemotherapy was monitored by multiparameter flow cytometry (FACSCanto Plus, BD Biosciences) of bone marrow samples, using one of two options of mAb combinations. One combination was CD11b-BV421 (BD Biosciences, ref. no. 562632; RRID:AB_2737689), CD13-APC (BD Biosciences, ref. no. 557454; RRID:AB_398624), CD33-PE-Cy7 (BD Biosciences, ref. no. 333952; RRID:AB_2713932), CD34-PerCP Cy5.5 (BioLegend, ref. no. 343522; RRID:AB_2228973), CD38-FITC (Beckman Coulter, ref. no. A07778; RRID:AB_2909810), CD45-V500 (BD Biosciences, ref. no. 647449; RRID:AB_2870319), CD117-PE (BD Biosciences, ref. no. 340529; RRID:AB_400044), and HLA-DR (BioLegend, ref. no. 307618; RRID:AB_493586). The other combination was CD7-APC (QuantoBio, ref. no. A6005R12; RRID:AB_2909814), CD14-APC-Alexa750 (Beckman Coulter, ref. no. B92421; RRID:AB_2909815), CD15-FITC (BD Biosciences, ref. no. 332778; RRID:AB_2868627), CD19-BV421 (BD Biosciences, ref. no. 562440; RRID:AB_11153299), CD33-PE-Cy7 (BD Biosciences, ref. no. 333952; RRID:AB_2713932), CD34-PE (QuantoBio, ref. no. Z6410008; RRID:AB_2910092), CD45-V500 (BD Biosciences, ref. no. 647449; RRID:AB_2870319), and CD56-PerCP Cy5.5 (BioLegend, ref. no. 318322; RRID:AB_893389).

### Targeted DNA Sequencing

Whole exons of a panel of 171 genes related to hematologic disorders (Supplementary Table S3) were selected for targeted next-generation sequencing (NGS). Genomic DNA from peripheral blood cells or bone marrow biopsy was isolated using QIAsymphony DSP DNA Mini Kit (QIAGEN) and then purified with AMPure XP Kit (Beckman Coulter). Coding exons were captured, enriched, proliferated, and purified using Twist Fast Hybridization and Wash Kit and Twist Binding and Purification Beads Kit (Twist Bioscience). DNA libraries were prepared using QIAseq FX DNA Library Kit (QIAGEN) and quantified by Quant-iT dsDNA HS Assay (Invitrogen). The amplicons were then paired-end sequenced on Illumina NovaSeq 6000 platform (Illumina, CA, USA; RRID:SCR_016387).

### Variant Calling after NGS

Sequencing artifacts or low-quality bases were discarded after quality control using FastQC (version 0.11.2; RRID:SCR_014583) and Trimmomatic (version 0.39; RRID:SCR_011848). Raw reads were then aligned to the reference human genome (UCSC GRCh37/hg19) using Burrows-Wheeler Aligner (version 0.7.10; RRID:SCR_010910). Variants were called using the following tools: SAMtools (version 0.1.19; RRID:SCR_002105) for bam file indexing, Picard (version 1.123; RRID:SCR_006525) for PCR duplicate elimination, GATK (version 3.5; RRID:SCR_001876) for marking insertion and deletion, and Pisces (version 5.1.6.54; https://github.com/Illumina/Pisces; RRID:SCR_022117) for single-nucleotide variant calling. ANNOVAR (RRID:SCR_012821) was then utilized to query each called variant against a few public databases: ESP6500si (RRID:SCR_012761), 1000 Genomes (RRID:SCR_008801), gnomAD (RRID:SCR_014964), ExAC (RRID:SCR_004068), and COSMIC (RRID:SCR_002260).

Germline samples were not available for most of the patients in THAMP. Somatic mutations were identified through the following pipeline: step 1, reject variants whose estimated probability of the base call being wrong was >0.01 (Q score < 20); step 2, reject variants where total sequencing depth (including wildtype and other variants) was <30 at the locus; step 3, reject variants whose variant allele fraction (VAF) was <0.02 or >0.45; step 4, reject variants that had appeared in healthy donors for bone marrow transplantation at the IHCAMS; step 5, rescue variants that had been rejected in steps 3 and 4 and yet appeared in the Catalogue of Somatic Mutations in Cancer (COSMIC) database (version 85) ≥2 times with the cancer loci recorded as “haematopoietic_and_lymphoid_tissue”; step 6, reject variants whose prevalence was >10% in the sequencing laboratory's previous runs on hematologic disease patients (interpreted to be false positives in our sequencing pipeline); step 7, reject synonymous mutations and intronic mutations; step 8, reject variants whose prevalence was >1% in any of the following public databases: ESP6500si, 1000 Genomes, gnomAD, or ExAC.

For the patients who carried mutations at CEBPA, those who carried more than one variants were considered “doubly mutated” (CEBPA-dm); the rest of the cases were considered “singly mutated” (CEBPA-sm).

FLT3-ITD was detected separately using a customized version of Genomon ITDetector (53). All positive cases underwent DNA fragment analysis, and then the allele ratio (AR; defined as the ratio of the area under the curve “FLT3-ITD” divided by area under the curve “FLT3-wild type”) was calculated. When AR was ≥0.5, the variant was classified as FLT3-ITD^high^; otherwise, it was classified as FLT3-ITD^low^.

### Experimental Validation of Variant Calls

Called somatic mutations with VAF ≥ 0.15 and >100x coverage were subjected to experimental validation by Sanger sequencing if both PCR primers and bone marrow DNA samples were readily available. The full list of validation experiments (covering 276 variant calls) is detailed in Supplementary Table S4. All of the primers had been designed using the web-based NCBI Primer-BLAST (https://www.ncbi.nlm.nih.gov/tools/primer-blast/; RRID:SCR_003095). PCR was performed using 200 ng DNA as the template. PCR products underwent agarose gel electrophoresis analysis and were subsequently purified with BigDye Terminator v3.1 Cycle Sequencing Kit (Applied Biosystems). Sanger sequencing of the purified amplicons was then conducted by capillary gel electrophoresis (ABI 3730 XL, Life Technologies).

### Dirichlet Process-Driven Clustering of Genetic Aberrations

Unlike previous single-disease studies that utilized Dirichlet process to cluster patients based on their genetic profiles (14, 30), we sought to cluster genetic aberrations based on their distributions across hematologic neoplasms. Suppose there were *p* genetic aberrations of interest and *q* diseases of interest. Suppose there were *n_j_* patients who were diagnosed with disease *j*, out of which *m_ij_* patients carried genetic aberration *i*. Suppose every gene in cluster *k* had true (unobserved) prevalence *θ_jk_* in disease *j*. Let *π_k_* be the number of genetic aberrations in cluster *k*. Let *C_i_* be the cluster label for genetic aberration *i*. We set up the following model:



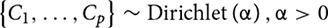





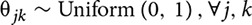





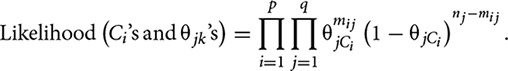



This model was then fitted via Markov chain Monte Carlo, i.e., reiterating between the following two steps:

1. Update *C_i_*: Suppose the latest value for *C_i_* was 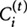. Sample a new value for *C_i_* according to

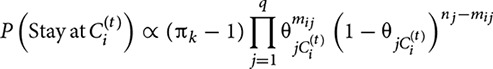

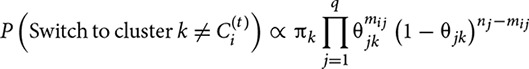

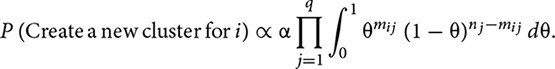
  Repeat this procedure for all *i*.
2. Update *θ_jk_*: Sample a new value for *θ_jk_* according to

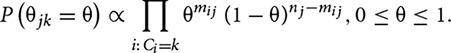

Repeat this procedure for all *j*, *k*.

We set the hyperparameter α = 0.0001. Model-fitting was initiated with each genetic aberration being its own standalone cluster. The burn-in period was 5,000 cycles, followed by 500 cycles of sampling from the posterior distribution. At the end of computation, each genetic aberration was assigned a cluster label that had the highest posterior probability.

### Two-Dimensional Embedding of the THAMP Patient Cohort

We trained a three-layered fully-connected neural network [input layer: 339 units; layer 1: 100 units with “rectified linear unit” (ReLU) activation; layer 2: 65 units with ReLU activation; layer 3: eight output units with softmax activation) using the “keras” application programming interface for deep learning to transform the raw phenogenetic data–including the mutational status at the 171 genes [174 variables (CEBPA mutations were classified into “CEBPA-sm” and “CEBPA-dm”, and FLT3 mutations were classified into “FLT3-ITD”, “FLT3-TKD (tyrosine kinase domain)”, and “FLT3-Other”)], presence/absence of the 72 chromosomal abnormalities, age, complete blood counts (36 variables), and peripheral blood/bone marrow aspiration smear analysis (56 variables)–into discrete diagnosis for each patient. After fitting the neural network, the output values of layer 2 were used as the input for principal component analysis (PCA) to embed each patient as a dot onto a two-dimensional phase space.

### Software Utilized

The neural network modeling was performed using “keras” (version 2.5.0) and “tensorflow” (version 2.5.0) in Python (RRID:SCR_008394). All the other computations were conducted with customized code in R (RRID:SCR_001905). R and Python code used in this study is available in a public GitHub repository at https://github.com/chenjunren-ihcams/THAMP (https://doi.org/10.5281/zenodo.6321600).

### Data Availability

The Chinese government approved the compilation of a desensitized THAMP dataset for facilitating international research collaborations on February 21^st^, 2022 [ref. no. CJ0273 (2022)] and subsequently approved the archiving of the dataset at the PRC National Genomics Data Center (NGDC) in April, 2022 (ref. no. 2022BAT1225). The THAMP dataset can be accessed at the NGDC website (https://ngdc.cncb.ac.cn/omix/release/OMIX001096/). The American Association of Cancer Research (AACR) Genomics Evidence Neoplasia Information Exchange (GENIE) 10.0-public gene mutation data (54) analyzed in this study were obtained from the AACR website (https://www.aacr.org/professionals/research/aacr-project-genie/aacr-project-genie-data/). The “Beat-AML-2018” data (55) analyzed in this study were obtained from the cBio Cancer Genomics Portal (cBioPortal) website (http://cbioportal.org/study/summary?id=aml_ohsu_2018).

## Results

### Benchmarking THAMP Against AACR GENIE 10.0-Public

Our gene panel “THAMP-Illumina-171” was a commercial panel (KingMed Diagnostics) routinely used for genotyping patients at initial diagnosis and relapse at the IHCAMS cancer clinics since February 2020. The panel originally contained 175 genes and was designed to provide 100% coverage of genes used in standard clinical practice of genetic risk stratification for hematologic neoplasms, along with an expanded set of additional genes selected based on literature search. Four of the genes in the panel (ARID1B, CRLF2, FBXO11, and KMT2C) did not pass quality control and were excluded from analysis; therefore, the final result contained mutational information regarding 171 genes (Supplementary Table S3). In our quality control experiments, 99.3% of the sequence variant calls in the THAMP dataset could be validated with Sanger sequencing (Supplementary Table S4).

We then benchmarked THAMP against AACR GENIE 10.0-public (54), an extensive database of gene mutations in cancer patients accessible at the AACR website (https://www.aacr.org/professionals/research/aacr-project-genie/aacr-project-genie-data/).

**FIGURE 1:**
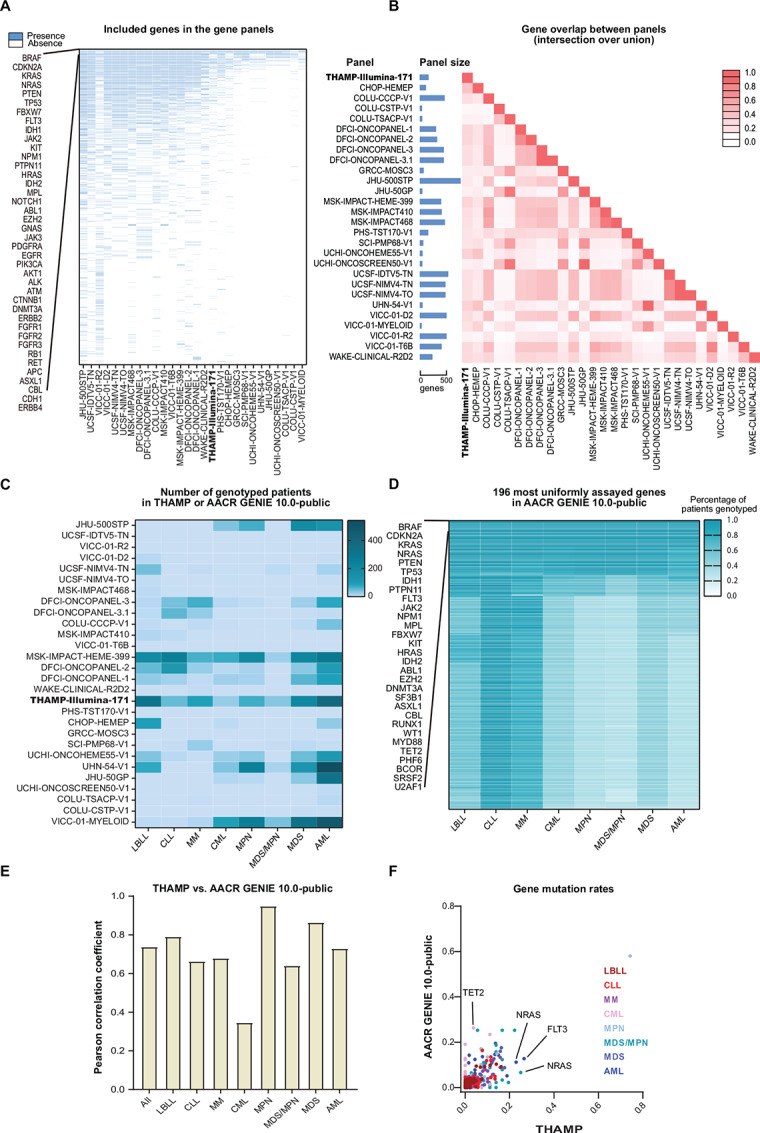
Benchmarking THAMP against AACR GENIE 10.0-public. **A,** Coverage of genes in THAMP-Illumina-171 and the 27 gene panels in AACR GENIE 10.0-public. **B,** Gene overlaps among the gene panels in THAMP and AACR GENIE 10.0-public. Overlap between each pair of the gene panels was calculated as the ratio between the number of genes in their intersection and the number of genes in their union. **C,** Distributions of patients across different diseases and across the compared gene panels in THAMP and AACR GENIE 10.0-public. ALL and lymphoblastic lymphoma were grouped together as LBLL in AACR GENIE 10.0-public. **D,** Genotyping percentages of the 196 most uniformly assayed genes across different diseases in AACR GENIE 10.0-public. **E,** Pearson correlation coefficients between the observed mutation rates of the 99 most uniformly assayed genes in THAMP and the observed mutation rates in AACR GENIE 10.0-public. **F,** Scatter plots comparing the observed mutation rates of the 99 most uniformly assayed genes in THAMP and the observed mutation rates in AACR GENIE 10.0-public.

A total of 27 gene panels (which covered 37 to 760 genes) were used for genotyping the 5,979 hematologic neoplasm cases [AML, *n* = 2,277; CLL, *n* = 515; CML, *n* = 327; lymphoblastic leukemia/lymphoma (LBLL), *n* = 592; MDS, *n* = 1076; MDS/MPN, *n* = 91; MM, *n* = 248; MPN, *n* = 853] in the AACR GENIE 10.0-public dataset, which was contributed by 13 research institutes [Children's Hospital of Philadelphia (CHOP; Philadelphia, PA); Columbia University (COLU; New York, NY); Dana-Farber Cancer Institute (DFCI; Boston, MA); Gustave Roussy Cancer Campus (GRCC; Paris, France); Johns Hopkins University (JHU; Baltimore, MD); Memorial Sloan Kettering Cancer Center (MSK; New York, NY); Providence Health & Services Cancer Institute (PHS; Portland, OR); Swedish Cancer Institute (SCI; Seattle, WA); University of Chicago Comprehensive Cancer Center (UCHI; Chicago, IL); University of California-San Francisco (UCSF; San Francisco, CA); Princess Margaret Cancer Centre-University Health Network (UHN; Toronto, Canada); Vanderbilt-Ingram Cancer Center (VICC; Nashville, TN); Wake Forest Baptist Medical Center (WAKE; Winston-Salem, NC)] (Fig. 1).

THAMP-Illumina-171 included 87% (26/30), 73% (29/40), and 68% (34/50) of the top 30, top 40, and top 50 most commonly assayed genes across all the panels, respectively (Fig. 1A; Supplementary Table S5). The average overlap of genes between THAMP-Illumina-171 and the 27 benchmark gene panels in AACR GENIE 10.0-public was 23% (range, 9% to 40%) (Fig. 1B). As a point of reference, the average overlap among the 27 benchmark gene panels in AACR GENIE 10.0-public was 27% (range, 4% to 100%); the union of all the 27 benchmark gene panels contained 1,498 genes, while the intersection contained merely six genes (BRAF, CDKN2A, KRAS, NRAS, PTEN, TP53). Since not all the cases in AACR GENIE 10.0-public were assayed with the same gene panel (Fig. 1C), most of the genes in AACR GENIE 10.0-public had quite uneven data collection across different diseases (Fig. 1D).

When we focused on the 99 most uniformly assayed (assayed in >90% of the patients in ≥1 diagnoses) genes (Supplementary Table S3) in the AACR GENIE 10.0-public dataset that overlapped with the 171 genes in THAMP, we found Pearson correlation coefficient of the observed mutation rates between the two datasets to be 0.74 overall and 0.81 if comparison was limited to AML, MDS, MDS/MPN, and MPN (Fig. 1E and F; Supplementary Table S6). (For each case in AACR GENIE 10.0-public, only the first entry (according to lexicographical sorting of SAMPLE_ID) of the sequencing data (interpreted to be the first time the case was sequenced) was utilized for mutation rate calculations.)

Despite the overall high concordance between THAMP and AACR GENIE 10.0-public, there were notable differences: First, CML had the lowest concordance between the two datasets (Fig. 1E). For example, while the mutation rate of TET2 (whose mutation was known to be more associated with advanced-phase CML than chronic-phase CML; refs. 56, 57) was 3.7% in CML in THAMP, it was 26.3% in CML in AACR GENIE 10.0-public (Fig. 1F). This could be due to difference in the timing of genotyping; while THAMP only included newly-diagnosed cases before treatment, the AACR GENIE 10.0-public dataset did not provide information related to disease phase. Second, while the mutation rate of NRAS in AML in THAMP (22.9%) was comparable to the numbers reported in other studies (58–60), it was considerably different from the observed mutation rate in AML in AACR GENIE 10.0-public (11.2%) (Fig. 1F).

### Clustering Genetic Aberrations According to their Prevalence Spectra

**FIGURE 2:**
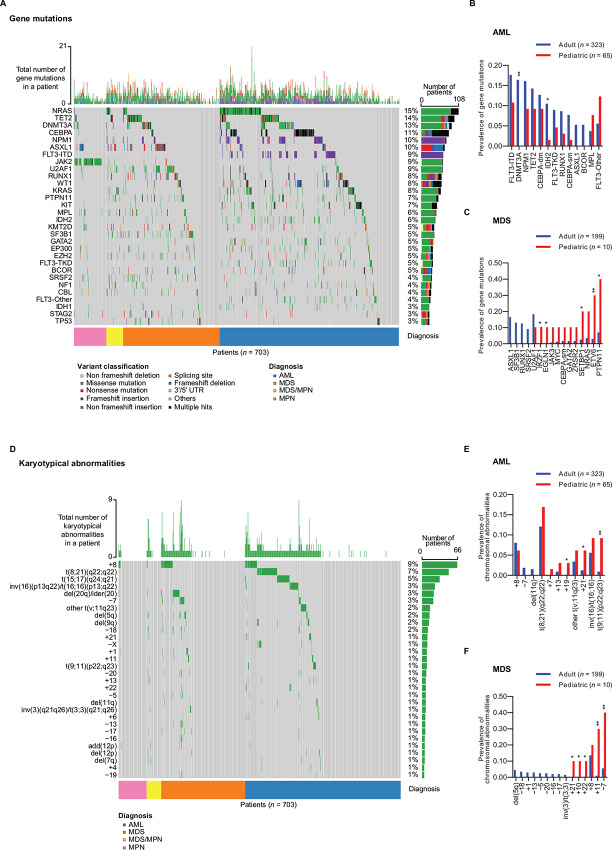
Observed spectra of gene mutations and karyotypical abnormalities in AML, MDS, MDS/MPN, and MPN in THAMP. **A,** Gene mutation spectra. “FLT3-Other” refers to FLT3 mutations that were neither FLT3-ITD nor FLT3-TKD. Age differences in gene mutation rates between the pediatric and adult cases for AML (**B**) and MDS (**C**). **D,** Karyotypical abnormality spectra. “Other t(v;11q23)” refers to KMT2A rearrangements other than t(9;11)(p22;q23). Age differences in karyotypical abnormality prevalence between the pediatric and adult cases for AML (**E**) and MDS (**F**). *, *P* < 0.05; **, *P* < 0.01. *P* values were calculated using the *χ*^2^ test (two-sided); no correction was performed to adjust for multiple-hypothesis testing. (AML: pediatric, *n* = 65; adult, *n* = 323 and MDS: pediatric, *n* = 10; adult, *n* = 199.)

In THAMP, the median number of observed gene mutations in a patient was 3, 3, 4, and 2 for AML, MDS, MDS/MPN, and MPN, respectively. The most commonly mutated genes were largely consistent with what has been reported in the literature, and they were–in descending order of overall mutation rate across the four diseases–NRAS (15%), FLT3 (15%), TET2 (14%), DNMT3A (13%), CEBPA (11%), NPM1 (10%), ASXL1 (10%), JAK2 (9%), U2AF1 (9%), RUNX1 (8%), WT1 (8%), KRAS (8%), PTPN11 (7%), KIT (7%), MPL (6%), IDH2 (6%), KMT2D (5%), SF3B1 (5%), and GATA2 (5%) (Fig. 2A).

Separate rankings of gene mutation rates for AML (Supplementary Fig. S1), MDS (Supplementary Fig. S2), MDS/MPN (Supplementary Fig. S3), and MPN (Supplementary Fig. S4) are available in Supplementary Data. The top five most prevalent gene mutations in AML were NRAS (23%), CEBPA (19%), FLT3-ITD (16%), NPM1 (15%), and DNMT3A (14%); in MDS they were U2AF1 (18%), TET2 (16%), ASXL1 (16%), DNMT3A (13%), and SF3B1 (12%); in MDS/MPN they were NRAS (25%), ASXL1 (22%), KRAS (19%), PTPN11 (17%), and SETBP1 (17%); finally, in MPN they were JAK2 (74%), ASXL1 (14%), CALR (10%), MPL (9%), and DNMT3A (7%).

We observed notable differences in gene mutation rates between the pediatric (age ≤16) and adult (age >16) cases in THAMP: For AML, FLT3-ITD and mutations in DNMT3A, NPM1, TET2, CEBPA, IDH2, FLT3-TKD, RUNX1, etc. were more prevalent in the adult cases, while non-ITD and non-TKD FLT3 mutations (“FLT3-Other”) and MPL mutations were more prevalent in the pediatric cases (Fig. 2B). Age differences in DNMT3A, NPM1, TET2, CEBPA, IDH2, RUNX1, and “FLT3-Other” for AML have been reported by Bolouri and colleagues recently (61). For MDS, mutations in ASXL1, SF3B1, RUNX1, SRSF2, etc. were more prevalent in the adult cases, while mutations in PTPN11, ETV6, NRAS, SETBP1, etc. were more prevalent in the pediatric cases (Fig. 2C). Age differences in ASXL1, SRSF2, PTPN11, NRAS, SETBP1, etc. for MDS have been reported by Schwartz and colleagues recently (62).

The most common cytogenetic abnormalities among AML, MDS, MDS/MPN, and MPN in THAMP were (in descending order of prevalence) +8 (9%), t(8;21)(q22;q22) (7%), t(15;17)(q24;q21) (5%), inv(16)(p13q22)/t(16;16)(p13;q22) (3%), t(v;11q23) (i.e., KMT2A rearrangements) (3%), del(20q)/ider(20) (3%), –7 (3%), del(5q) (2%), del(9q) (2%), –18 (2%), +21 (1%), –X (1%), +1 (1%), +11 (1%), –20 (1%), +13 (1%), +22 (1%), –5 (1%), del(11q) (1%), and inv(3)(q21q26)/t(3;3)(q21;q26) (1%) (Fig. 2D and Supplementary Table S7). Many of these prevalent chromosomal abnormalities [e.g., +8, t(8;21)(q22;q22), inv(16)(p13q22)/t(16;16)(p13;q22), t(v;11q23), del(20q)/ider(20), –7, del(5q), −5, del(11q), inv(3)(q21q26)/t(3;3)(q21;q26)] are listed verbatim in the 2017 ELN and IPSS-R risk stratification schemes, while the others indirectly contribute to risk stratification through being counted as constituents of “complex karyotypes”.

Separate rankings of karyotypical abnormalities for AML (Supplementary Fig. S1), MDS (Supplementary Fig. S2), MDS/MPN (Supplementary Fig. S3), and MPN (Supplementary Fig. S4) are available in Supplementary Data. The top five most prevalent karyotypical abnormalities in AML were t(8;21)(q22;q22) (13%), t(15;17)(q24;q21) (9%), +8 (8%), inv(16)(p13q22)/t(16;16)(p13;q22) (6%), and t(v;11q23) (6%); in MDS they were +8 (14%), del(20q)/ider(20) (9%), –7 (7%), del(5q) (4%), and –18 (3%); in MDS/MPN they were +13 (6%), +8 (6%), –18 (6%), del(5q) (6%), and del(7q) (6%); finally, in MPN they were +8 (7%), +1 (1%), –6 (1%), add(12p) (1%), and del(20q)/ider(20) (1%).

We observed notable differences in karyotypical abnormality prevalence between the pediatric (age ≤16) and adult (age >16) cases in THAMP: For AML, t(v;11q23), inv(16)(p13q22)/t(16;16)(p13;q22), +21, +19, etc. were more prevalent in the pediatric cases (Fig. 2E). Age differences in t(v;11q23) and inv(16) for AML were also reported by Bolouri and colleagues recently (61). For MDS, –7, +11, etc. appeared to be more prevalent in the pediatric cases (Fig. 2F). Relative prevalence of –7 in pediatric MDS has been reported by Schwartz and colleagues recently (62).

**FIGURE 3:**
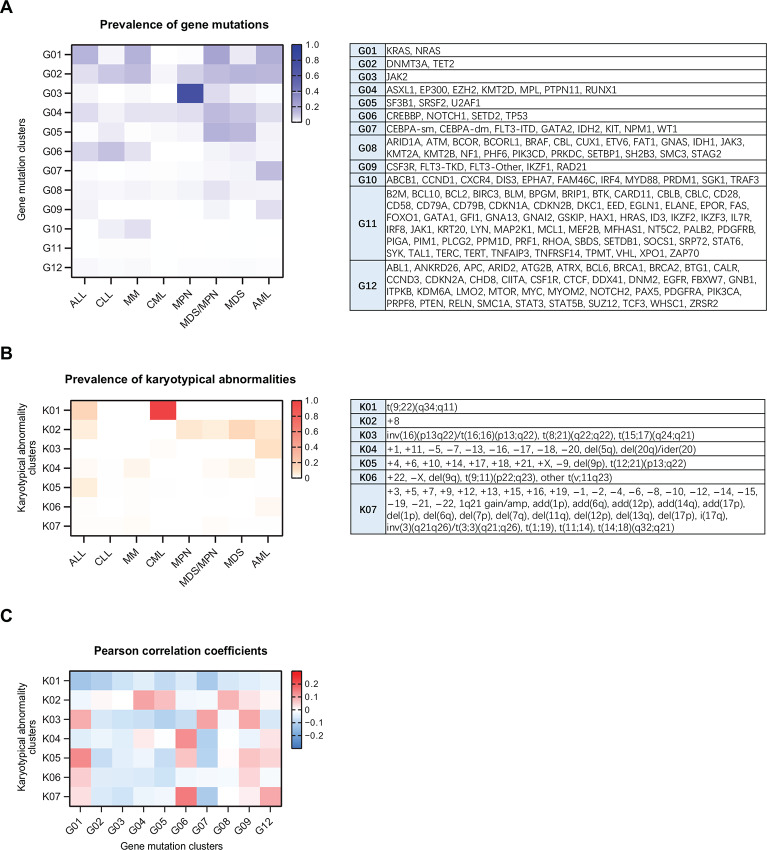
Dirichlet process-driven clustering of gene mutations and karyotypical abnormalities in THAMP. **A,** Gene mutations. **B,** Karyotypical abnormalities. **C,** Pearson correlation coefficients between the gene mutation clusters and the karyotypical abnormality clusters across all the eight diseases.

A Dirichlet process-based Markov chain Monte Carlo algorithm was applied to THAMP and classified the 171 genes into 12 clusters according to their mutational spectra (Fig. 3A). Gene mutation clusters G01, G02, G04, G08, and–to a lesser extent–G09 were relatively prevalent in both myeloid and lymphoid neoplasms. In contrast, the other clusters were skewed towards one of the two lineages: mutations in clusters G06 and G10 were in general more associated with lymphoid neoplasms than myeloid neoplasms; on the other hand, mutations in clusters G03, G05, and G07 were more associated with myeloid neoplasms than lymphoid neoplasms.

Clustering of karyotypical abnormalities was also conducted, resulting in seven clusters of chromosomal abnormalities (Fig. 3B). As expected, cluster K01 [which contained one sole abnormality t(9;22)(q34;q11), the cytogenetic signature for the BCR/ABL1 fusion gene] was primarily associated with CML and to a lesser extent ALL. Clusters K03 and K06 (which contained many of the cytogenetic abnormalities listed in the 2017 ELN risk stratification scheme for AML) was associated with AML primarily. Likewise, cluster K04 (which contained many of the cytogenetic abnormalities listed in the IPSS-R risk stratification scheme for MDS) was associated with MDS primarily. Cluster K05 [which contained the cytogenetic signature t(12;21)(p13;q22) for the TEL/AML1 fusion gene, a common genetic aberration in ALL] was associated with ALL primarily. Finally, cluster K02 (which contained one sole abnormality +8) was associated with ALL and most of the myeloid neoplasms.

We calculated Pearson correlation coefficients between the gene mutation clusters and the karyotypical abnormality clusters across the eight diseases (Fig. 3C). The magnitude of correlations was small (ranging from −0.12 to 0.15), but there were distinct patterns. For instance: gene mutation clusters G04 and G08 (associated with both myeloid and lymphoid neoplasms) and karyotypical abnormality cluster K02 (associated with both myeloid and lymphoid neoplasms) were positively correlated; gene mutation cluster G07 (associated more with myeloid neoplasms) was positively correlated with karyotypical abnormality cluster K03 (associated more with myeloid neoplasms); finally, gene mutation clusters G01 and G09 (associated with both myeloid and lymphoid neoplasms) was positively correlated with karyotypical abnormality cluster K03 (associated more with myeloid neoplasms) and K05 (associated more with lymphoid neoplasms).

### Correlations between Gene Mutations and Phenotypic Features in Myeloid Neoplasms

**FIGURE 4:**
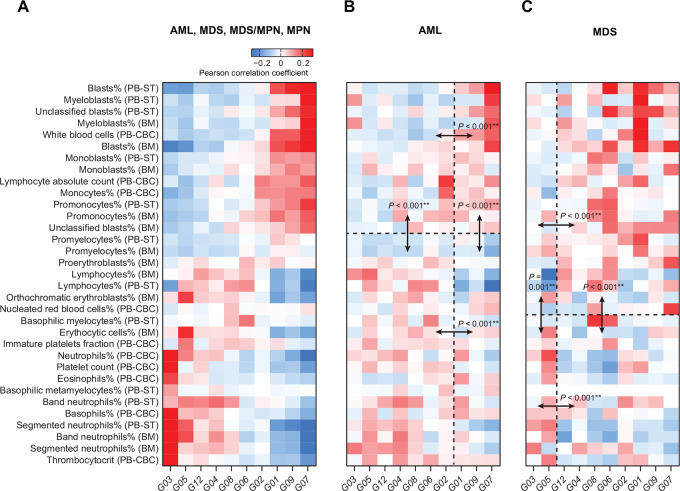
Pearson correlation coefficients between gene mutations and phenotypic features in myeloid neoplasms according to the THAMP dataset. **A,** All myeloid neoplasms (AML, MDS, MDS/MPN, MPN). **B,** AML. **C,** MDS. Block comparisons were conducted in (**B)** and (**C)** to highlight statistically significant differences between the distributions of Pearson correlation coefficients in the adjacent blocks; *P* values were calculated using the Wilcoxon test (two-sided). Without affecting any analytical result, the phenotypic features are plotted in the same order as in Fig. 5E, and the gene mutation clusters are plotted in the same order as in Fig. 6A. PB, peripheral blood; BM, bone marrow; CBC, complete blood count; ST, smear test. **, *P* < 0.01.

Since we were interested in characterizing the phenogenetic patterns along the entire myeloid neoplasm continuum in this study, we also calculated Pearson correlation coefficients between gene mutations and a wide range of phenotypic features across AML, MDS, MDS/MPN, and MPN (Fig. 4A).

Interestingly, we found that there was a prominent gradient of phenogenetic correlations between two extremes of clinical manifestations. Reading Fig. 4A from top to bottom: blast counts (in both bone marrow and peripheral blood) and white blood cell count (peripheral blood) were prominently positively correlated with the total number of mutations in cluster G07 and (to a lesser extent) clusters G09 and G01—consistent with previously reported associations in CEBPA, CSF3R, FLT3, GATA2, IDH, KIT, NPM1, and WT1 (6, 37–43). This was followed by lymphocyte count (peripheral blood), monocyte percentage (peripheral blood), and promonocyte percentage (bone marrow), which were the most positively correlated with gene mutation clusters G07, G09, G01, and G02; previously, GATA2 mutation (classified as G07) has been reported to be correlated with monocytosis (46). Then, lymphocyte percentage (peripheral blood) and basophilic myelocyte percentage (peripheral blood) were positively correlated with clusters G06 and G08. Orthochromatic erythroblast percentage (bone marrow), erythrocytic cell percentage (bone marrow), immature platelet fraction (peripheral blood), etc. were correlated with cluster G05; previously, SF3B1 mutation (classified as G05) has been reported to be positively correlated with high platelet count (44). Finally, neutrophil percentage (peripheral blood), platelet count (peripheral blood), eosinophil percentage (peripheral blood), band neutrophil percentage (bone marrow), etc. were the most positively correlated with gene mutations in cluster G03. We also observed prominent negative correlations between platelet count (peripheral blood) and mutations in clusters G07, G09, G01, and G06; previously, mutations in TP53 (classified as G06) and NRAS (classified as G01) have been reported to be associated with severe thrombocytopenia (47).

We also investigated if the global-scale correlation between clinical manifestations and gene mutations was spurious due to Simpson Paradox, wherein an observed global pattern might disappear or even become reversed when analysis was limited to a subgroup of patients (63). If Simpson Paradox was in effect, we anticipated that the phenogenetic correlation we observed on the global scale in Fig. 4A would cease to stand when we focused on a subset of the patients who were diagnosed with the same disease.

Keeping the same order of phenotypic features and the same order of gene mutation clusters as in Fig. 4A, we found that even when we limited our correlation analysis to AML only, phenotypic features at the top tended to be more correlated with gene mutation clusters on the right, while phenotypic features at the bottom tended to be more correlated with gene mutation clusters on the left (Fig. 4B). Similar phenomenon could also be observed in MDS (Fig. 4C). Therefore, global-scale correlations between phenotypic features and gene mutations were also reflected locally, i.e., in subgroups of patients.

### Computation of the Pan-Myeloid Axis through Integration of Multidimensional Phenogenetic Data

**FIGURE 5:**
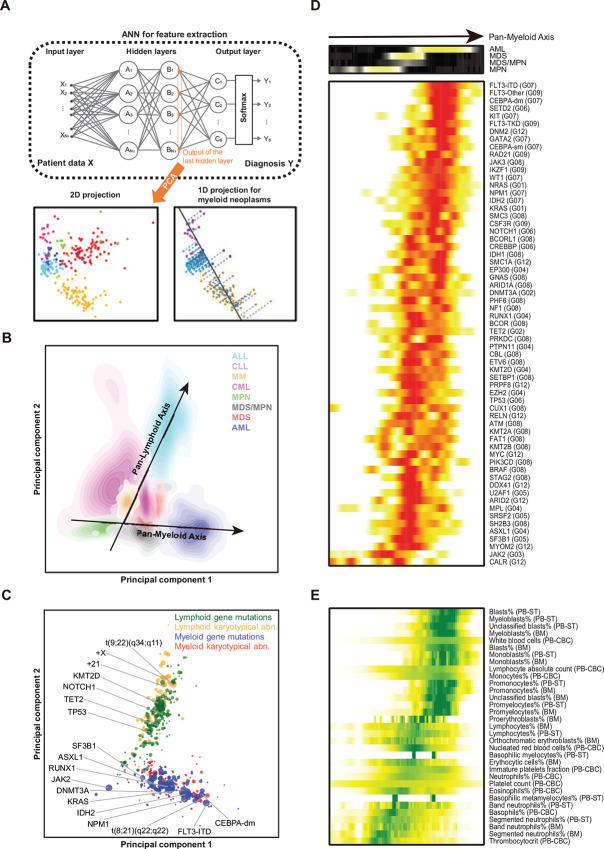
Computing the Pan-Myeloid Axis based on the THAMP dataset. **A,** Diagram for the computational pipeline. **B,** Two-dimensional representation of all the patients in THAMP. **C,** Centroid locations of genetic aberrations in the two-dimensional phase space. abn., abnormalities. **D,** Mutation rates of selected genes along the Pan-Myeloid Axis (the full diagram that includes all the 171 genes in THAMP-Illumina-171 is available in Supplementary Fig. S5). **E,** Average profiles of selected phenotypic features along the Pan-Myeloid Axis. The full diagram that includes all the 92 phenotypic features in THAMP-Illumina-171 is available in Supplementary Fig. S6.

To further understand how genetic and phenotypic aberrations jointly related to disease diagnosis, an artificial neural network (ANN; Fig. 5A) was fitted to the complete patient dataset that comprised mutational status at the 171 genes (174 variables), presence/absence of 72 chromosomal abnormalities, age, complete blood counts (36 variables), and peripheral blood/bone marrow aspiration smear analysis (56 variables; Supplementary Table S8). The area under the ROC curve (AUROC) of the ANN model reached a weighted average of 0.92 (ALL, 0.95; AML, 0.92; CLL, 0.90; CML, 0.86; MDS, 0.91; MDS/MPN, 0.73; MM, 0.93; MPN, 0.88) in five-fold cross-validation, indicating that the model indeed captured high-dimensional relationships among genetic and phenotypic aberrations. The output values at the last hidden layer in the ANN (interpreted as learned low-dimensional nonlinear representations of the patients’ multidimensional/multimodal clinical and genetic profiles) were then used to project all the patients onto a two-dimensional phase space via PCA to visualize how the 1,192 patients related to each other phenogenetically (Fig. 5A and B).

We found that the myeloid and lymphoid cases in THAMP were distinctly grouped into two diverging axes, which we termed the “Pan-Myeloid Axis” and the “Pan-Lymphoid Axis” (Fig. 5B). Interestingly, CML formed a distinct group by itself, apart from the Pan-Myeloid and Pan-Lymphoid axes; this was consistent with the long-known fact that CML–despite being a myeloid neoplasm–in blast crisis can sometimes transform to ALL, a lymphoid cancer. When we projected onto the two-dimensional phase space the center of probabilistic mass of each gene mutation and each karyotypical abnormality (Fig. 5C), we found that–consistent with Fig. 3–the Pan-Myeloid and the Pan-Lymphoid axes each had its associated prevalent genetic aberrations, and they appeared to lie in particular orders along the two axes.

To further characterize AML, MDS, MDS/MPN, and MPN in THAMP, we projected the patients diagnosed with these four diseases onto the one-dimensional “Pan-Myeloid Axis” (Fig. 5A). When “walking” from the left (“MDS-ish”) end of the Pan-Myeloid Axis towards its right (“AML-ish”) end, gene mutations shifted from cluster G03; then clusters G05, G12, G04, and G08; then clusters G06 and G02; and finally clusters G01, G09, and G07 (Fig. 5D; Supplementary Fig. S5). Phenotypic features also progressed continuously from one end of the Pan-Myeloid Axis to the other end (Fig. 5E; Supplementary Fig. S6).

### Simplifying Gene Mutation Groupings for the Pan-Myeloid Axis

**FIGURE 6:**
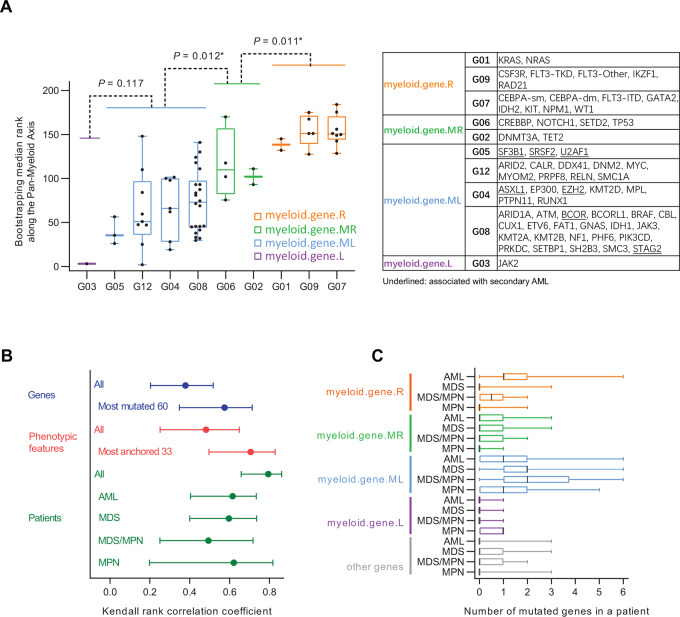
Simplifying gene mutation groupings for the Pan-Myeloid Axis. **A,** Regrouping of gene mutations. *P* values were calculated using the Wilcoxon test (two-sided). **B,** Stability analysis of the orders of patients, gene mutations, and phenotypic features along the Pan-Myeloid Axis through bootstrapping experiments. Shown here are mean values and 95% confidence intervals (CI). The bootstrapping experiment was performed independently for 100 times. Stability was evaluated by calculating the values of Kendall rank correlation coefficient for all possible pairs of bootstrapping results. **C,** Distributions of gene mutations in groups “myeloid.gene.R” (12 genes), “myeloid.gene.MR” (6 genes), “myeloid.gene.ML” (41 genes), “myeloid.gene.L” (1 gene), and other genes (111 genes) in various myeloid neoplasms in THAMP. Box plots indicate minimum, maximum, and the 25th, 50th, and 75th percentile values. *, *P* < 0.05.

The gene mutation clustering reported in Fig. 3A was calculated using all the 1,192 patients. Since we were primarily interested in myeloid neoplasms in this study, in light of the new information generated by the one-dimensional rendering of myeloid neoplasms (Fig. 5), we further simplified gene mutation clustering for myeloid neoplasms by designating cluster G03 as “myeloid.gene.L”; grouping G05, G12, G04, and G08 genes that had >1% mutation rate in the myeloid neoplasms in THAMP into “myeloid.gene.ML”; grouping G06 and G02 genes that had >1% mutation rate in the myeloid neoplasms into “myeloid.gene.MR”; and grouping G01, G09, and G07 genes that had >1% mutation rate in the myeloid neoplasms into “myeloid.gene.R” (Fig. 6A). All the other genes were designated as “other genes”.

Using bootstrapping experiments, we confirmed that the orders of genes, phenotypic features, and patients along the Pan-Myeloid Axis were stable with statistical significance (Fig. 6B). In other words, the patterns we reported in Fig. 5D and E, and Fig. 6A were not dependent on one particular composition of patients and therefore less likely to be spurious discoveries.

Note that the ranking order of cancer genes along the Pan-Myeloid Axis was not a naïve sorting of genes based on their mutation rates in myeloid neoplasms (Fig. 6C). While the average mutation rate of genes in AML indeed formed a decrescendo from the right end of the Pan-Myeloid Axis to its left end (“myeloid.gene.R”, 10.1% in AML; “myeloid.gene.MR”, 5.8% in AML; “myeloid.gene.ML”, 2.6% in AML; “myeloid.gene.L”, 1.0% in AML; “other genes”, 0.3% in AML), mutation rates of individual genes were a lot more scrambled. For instance, CSF3R, IKZF1, and RAD21 were in group “myeloid.gene.R”, and yet their mutations rates in AML were lower than the average of “myeloid.gene.MR”. Similarly, PTPN11 and RUNX1–both in “myeloid.gene.ML”–had higher mutation rates in AML than the average of “myeloid.gene.MR”.

### Clinical Relevance of the Pan-Myeloid Axis in Adult *De Novo* AML

We subsequently asked if the Pan-Myeloid Axis we uncovered in Figs. 5 and 6 had any relevance to treatment response, even though no clinical outcome data had been used in any computation so far. The ideal experiment to truly answer how clinical course varies along the Pan-Myeloid Axis would be administering the same array of treatment regimens to more than one disease along the axis—similar to what was recently proposed by Estey and colleagues (5). Only through such clinical trials can one determine which patients should be treated with “AML-type” regimens, which patients should be treated with “MDS-type” regimens, etc. In the real world, however, most patients are treated according to their diagnoses based on the contemporary diagnostic criteria; thus, patients with “AML” are usually treated with “AML-type” regimens, and patients with “MDS” are usually treated with “MDS-type” regimens. In other words, the clinical course of a patient cannot be evaluated independently of diagnostic criteria. Still, it would have been a missed opportunity to not investigate if the Pan-Myeloid Axis could also help with risk stratification in a single disease. Our reasoning was that some of the patients might not respond well to standard treatment regimens because they deviated from the canonical cases, and one evidence for this could be that they were situated at some distance from the canonical cases on the Pan-Myeloid Axis.

We first examined the AML cases in THAMP (Supplementary Table S9). To eliminate confounding influence from age and the PML-RARA fusion gene (which has favorable prognosis and is usually treated with a chemotherapy regimen that is entirely different from other AML subtypes; refs. 64, 65), we limited clinical outcome analysis to the adult (age >16) *de novo* cases that were not M3. Since the patients with AML in THAMP were all relatively recent, our analysis focused on MRD after induction therapy.

Among the 247 adult non-M3 *de novo* AML cases in THAMP that had MRD data, 204 (83%) were treated with the standard “anthracycline + cytarabine” induction therapy, 28 (11%) received hypomethylating agents, 3 (1%) received Venetoclax, 4 (2%) received both Venetoclax and hypomethylating agents, and the rest [8 (3%)] were treated with other induction regimens. We focused our analysis on the 204 adult non-M3 *de novo* AML cases who were treated with the standard induction therapy.

**FIGURE 7:**
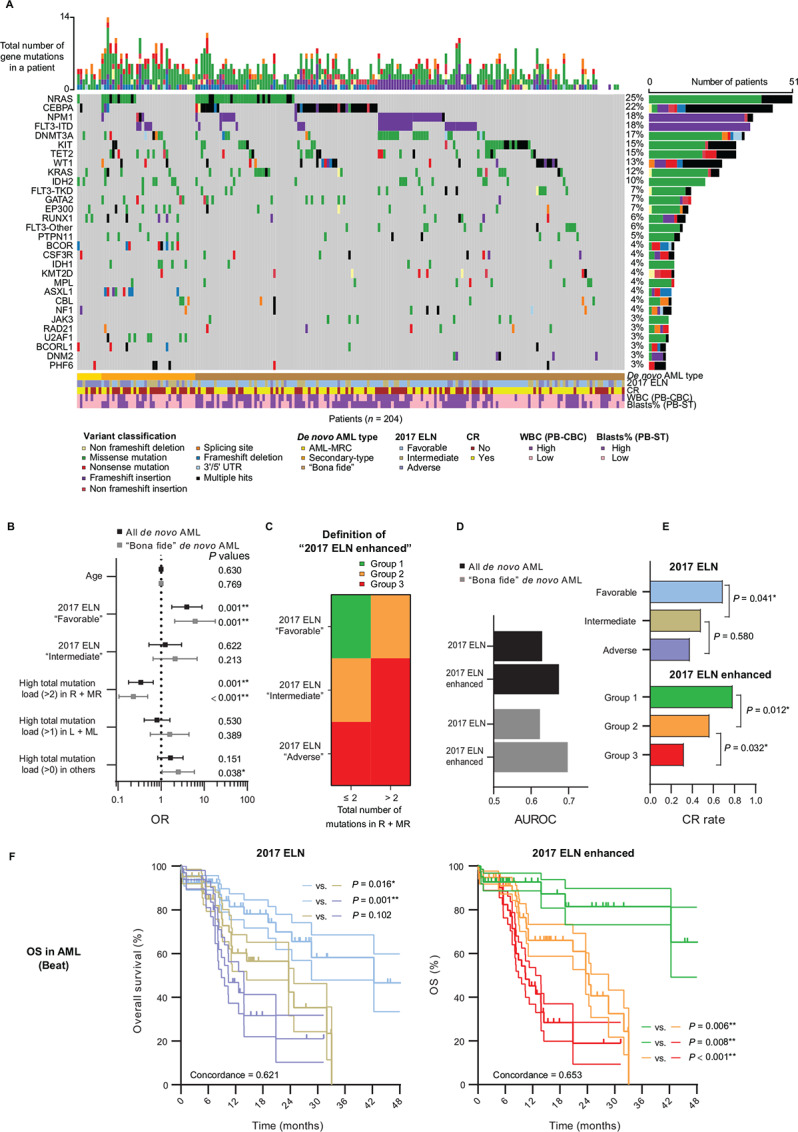
Clinical relevance of the Pan-Myeloid Axis for adult *de novo* non-M3 AML. **A,** Gene mutation spectra of adult AML-MRC, “secondary-type”, and “bona fide” *de novo* AML (with M3 excluded) in THAMP. **B,** Multivariate analysis for predicting CR in THAMP. The fitted model was logistic regression. *P* values were calculated using the Wald test (two-sided). The 60th percentile values of the total number of mutations in “R + MR”, “L + ML”, and “others” in all the adult patients with *de novo* non-M3 AML were 2, 1, and 0, respectively (all *de novo* AML, *n* = 204; “bona fide” *de novo* AML, *n* = 160). **C,** The proposed “2017 ELN enhanced” risk stratification scheme. **D,** AUROCs for predicting CR in THAMP. Only one trial was conducted in each scenario; therefore, there was no standard error (SE) or CI. **E,** CR rates for different strata of the patients with “bona fide” *de novo* AML in THAMP. *P* values were calculated using the Fisher exact test (two-sided). 2017 ELN: “Favorable”, *n* = 104 [66 (63%) and 38 (37%) were reclassified by “2017 ELN enhanced” as “Group 1” and “Group 2”, respectively]; “Intermediate”, *n* = 35 [22 (63%) and 13 (37%) were reclassified by “2017 ELN enhanced” as “Group 2” and “Group 3”, respectively]; “Adverse”, *n* = 21 (all were classified by “2017 ELN enhanced” as “Group 3”). 2017 ELN enhanced: “Group 1”, *n* = 66; “Group 2”, *n* = 60; “Group 3”, *n* = 34. **F,** Kaplan–Meier curves and respective SEs for different strata of the patients with “bona fide” *de novo* AML in the Beat-AML-2018 dataset. *P* values of pairwise comparisons were calculated using the Wald test (two-sided) in Cox regression. 2017 ELN: “Favorable”, *n* = 65 [41 (63%) and 24 (37%) were reclassified by “2017 ELN enhanced” as “Group 2” and “Group 3”, respectively]; “Intermediate”, *n* = 42 [33 (79%) and 9 (21%) were reclassified by “2017 ELN enhanced” as “Group 2” and “Group 3”, respectively]; “Adverse”, *n* = 33 (all were classified by “2017 ELN enhanced” as “Group 3”). 2017 ELN enhanced: “Group 1”, *n* = 41; “Group 2”, *n* = 57; “Group 3”, *n* = 42. *, *P* < 0.05; **, *P* < 0.01.

We recognized that even these relatively homogeneous adult *de novo* non-M3 AML cases could yet still be more heterogeneous than ideal. For instance, we were aware of at least two additional confounding factors that could interfere with our analysis: First, 9 (4%) of the remaining 204 *de novo* AML cases carried myelodysplastic-related changes (“AML-MRC”); they could be secondary AML cases in reality, but somehow their MDS prodromes had escaped diagnosis (21). Second, 35 (17%) additional cases carried gene mutations (SRSF2, SF3B1, U2AF1, ZRSR2, ASXL1, EZH2, BCOR, or STAG2) that were commonly associated with secondary AML (“secondary-type AML”; ref. 13), even though they did not carry myelodysplastic-related changes according to the current WHO criteria. Close examination of these two subgroups of the patients showed that their gene mutational spectra and phenotypic features were indeed substantially different from the other adult *de novo* non-M3 AML (“bona fide”) cases (Fig. 7A).

Regardless of whether we excluded the AML-MRC and secondary-type AML cases from analysis or not, we found that high mutation load in gene groups “R + MR” (defined as carrying >2 mutations in “myeloid.gene.R” and “myeloid.gene.MR” combined) added independent prognostic value for predicting complete remission (CR) after induction (defined as MRD < 0.1% according to flow cytometry analysis of bone marrow samples) even when age and the 2017 ELN genetic risk score were included as covariates in the multivariate analysis (Fig. 7B).

Moreover, integration of the 2017 ELN genetic risk scoring scheme and the relational information of genes along the Pan-Myeloid Axis allowed us to reliably divide adult patients with *de novo* non-M3 AML into three subgroups that had better separation of CR rates than using the 2017 ELN risk scores alone (Fig. 7C–E): Group 1, “2017 ELN Favorable and low total mutation load (≤2) in R + MR”; Group 2, “2017 ELN Favorable and high total mutation load (>2) in R + MR” or “2017 ELN Intermediate and low total mutation load (≤2) in R + MR”; and Group 3, “2017 ELN Intermediate and high total mutation load (>2) in R + MR” or “2017 ELN Adverse”. (Gene mutations that were already counted in “2017 ELN” were counted again when calculating the total mutation load in “R + MR”. In other words, computations along the two dimensions in Fig. 7C did not interfere with each other.) We termed this new risk scoring system “2017 ELN enhanced”. In particular, AUROC for predicting CR increased from 0.62 (2017 ELN) to 0.70 (2017 ELN enhanced) when we focused on the “bona fide” *de novo* cases (Fig. 7D).

To validate the proposed “2017 ELN enhanced” scoring system and also to further investigate the prognostic significance of the Pan-Myeloid Axis, we queried cBioPortal in search of *de novo* AML patient sequencing data that included survival information. There was one hit–referred to as the “Beat-AML-2018” dataset (55) in this study–that contained 140 adult (age >16) patients with non-M3 *de novo* AML who had clearly defined 2017 ELN genetic risk scores, had disease samples sequenced at initial diagnosis, were not recorded as AML-MRC, did not carry “secondary-type” gene mutations, were treated with standard chemotherapy, and had known overall survival (OS) status.

Applying the same “2017 ELN enhanced” risk scoring method described in Fig. 7C, we found that the three subgroups of patients with *de novo* AML had mutually distinct survival curves that passed statistical significance, with much better intersubgroup separations than 2017 ELN risk stratification (Fig. 7F). In addition, the concordance score for predicting OS improved from 0.62 (2017 ELN) to 0.65 (2017 ELN enhanced).

Therefore, although the Pan-Myeloid Axis was discovered through a cross-disease analysis that was meant to quantitatively characterize the transitions between the different diseases, this axis nonetheless carried statistically significant information related to how the patients with “*de novo* AML” who were diagnosed with the current diagnostic criteria responded to standard “AML-type” treatment.

### Clinical Relevance of the Pan-Myeloid Axis in Adult MDS-EB

**FIGURE 8:**
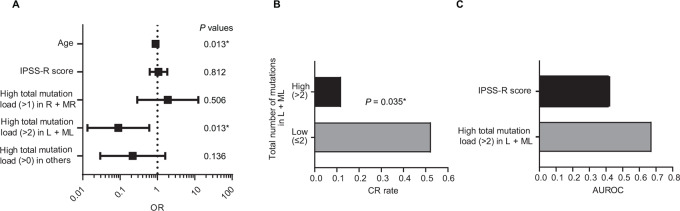
Clinical relevance of the Pan-Myeloid Axis for adult MDS-EB. **A,** Multivariate analysis for predicting complete remission in THAMP. The fitted model was logistic regression (*n* = 42). *P* values were calculated using the Wald test (two-sided) in logistic regression. The 60th percentile values of the total number of mutations in “R + MR”, “L + ML”, and “others” in the adult MDS-EB patients were 1, 2, and 0, respectively. **B,** CR rates for the patients with MDS-EB stratified according to the total mutation load in “L + ML” [“High” (>2 mutations), *n* = 13; “Low” (≤2 mutations), *n* = 29]. *P* values were calculated using the Fisher exact test (two-sided). **C,** AUROCs for predicting CR in THAMP. Only one trial was conducted for each stratification model; therefore, there was no SE or CI. *, *P* < 0.05; **, *P* < 0.01.

Finally, we examined the MDS cases in THAMP (Supplementary Table S10) to investigate if there was also evidence for correlation between the Pan-Myeloid Axis and treatment response in MDS. 42 adult patients (age >16) of MDS with excess blasts (MDS-EB) in THAMP underwent chemotherapy that included both hypomethylating agents and cytotoxic drugs. CR (defined as ≤5% myeloblasts in bone marrow nucleated cells and decrease by ≥50% over pretreatment) after six cycles of chemotherapy was used as the criterion for treatment response. We found that high mutation load in “L + ML” (defined as carrying >2 mutations in “myeloid.gene.L” and “myeloid.gene.ML” combined) was significantly correlated with CR in MDS-EB (Fig. 8A and B). In particular, AUROC increased from 0.42 (based on IPSS-R) to 0.67 (based on the total mutation load in “L + ML”) (Fig. 8C). Thus, the Pan-Myeloid Axis carried statistically significant information related to how the patients with “MDS-EB” who were diagnosed with the current diagnostic criteria responded to standard “MDS-EB–type” treatment.

## Discussion

We acknowledge several limitations in our study: First, the THAMP cohort only contained patients that were treated at a single medical center; therefore, conclusions of this study might not extrapolate to other patient groups. Second, our study cohort was composed of primarily patients of Han ethnicity (self-identified ethnicities: Han, *n* = 1,105; Manchu, *n* = 37; Mongol, *n* = 26; Hui, *n* = 16; Korean, *n* = 3; Kazakh, *n* = 2; Uyghur, *n* = 1; Tibetan, *n* = 1; unidentified, *n* = 1), and our observed spectra of genetic aberrations might be specific to this particular composition of racial backgrounds, as interracial differences have been reported previously (66–68). Third, our study of mutational spectra was based on targeted sequencing and might have missed crucial driver mutations or concomitant mutations. Fourth, the patients with AML and MDS in THAMP were primarily treated with “AML-type” and “MDS-type” regimens, respectively, and therefore we were limited to examining the clinical impact of the computed Pan-Myeloid Axis “locally” (i.e., examining how the axis modulates AML and MDS patients’ response to standard AML and MDS therapies, respectively), rather than “globally” (i.e., examining how the axis modulates the responses of a continuum of patients with myeloid neoplasm to an array of commonly-administered therapy regimens; refs. 5, 22, 28). Fifth, our data did not allow us to examine the temporal order of mutations, which had been reported to be influential on phenotype, disease course, and treatment outcome in myeloid neoplasms (69–72). Sixth, we did not have preclinical molecular data for the THAMP patients; as such, we did not investigate how CHIP affected these patients’ trajectories of clinical manifestations and disease progress, a subject that has received more attention lately (24, 25, 73). Seventh, the THAMP cohort was very recent, and our study was limited by its relatively short follow-up period.

Nonetheless, our study had the advantage that all the patients were subjected to the same protocols for genotypic and phenotypic data collection. Our comparative genomics analysis provided strong evidence for the existence of a phenogenetically-defined “Pan-Myeloid Axis”, by which commonly mutated genes in myeloid neoplasms were classified into right (“AML-ish”) versus left (“MDS-ish”). Other than FLT3-ITD and TP53 (both of which have already been incorporated as adverse genetic factors in the 2017 ELN risk stratification scheme; ref. 17), our study in addition affirmed adverse prognosis of mutations in other genes such as CSF3R, DNMT3A, IDH2, KIT, KRAS, NOTCH1, NRAS, and TET2 (all classified as “myeloid.gene.R” or “myeloid.gene.MR”) in adult *de novo* non-M3 AML, consistent with some recent publications (38, 40, 74–78).

We observed some correlations between the Pan-Myeloid Axis and gene function. For instance, numerous leukemic genes involved in receptor tyrosine kinase pathways such as FLT3, KIT, KRAS, and NRAS (all classified as “myeloid.gene.R”) were situated at the far right of the Pan-Myeloid Axis, while preleukemic genes such as TET2 (classified as “myeloid.gene.MR”), DNMT3A (classified as “myeloid.gene.MR”), and ASXL1 (classified as “myeloid.gene.ML”) that were associated with CHIP (79–82) were all placed more to the left. In addition, all the gene mutations that have been previously reported to be associated with secondary AML (SRSF2, SF3B1, U2AF1, ZRSR2, ASXL1, EZH2, BCOR, or STAG2; ref. 13) were all classified as “myeloid.gene.ML” and situated at the left side of the Pan-Myeloid Axis. Note, however, that the “2017 ELN enhanced” risk stratification scheme continued to perform substantially better than the 2017 ELN scheme even when all the AML-MRC and “secondary-type” AML cases were excluded from our clinical outcome analysis (to be more precise, “2017 ELN enhanced” exhibited even stronger advantage over “2017 ELN” after we eliminated the AML-MRC and “secondary-type” AML cases). Therefore, the Pan-Myeloid Axis was not a mere recapitulation of the molecular distinction between *de novo* AML and secondary AML.

There were apparent discrepancies between the THAMP-defined Pan-Myeloid Axis and the 2017 ELN genetic risk stratification scheme. Most notably, while NPM1 mutation was labeled as favorable in 2017 ELN (especially when FLT3-ITD allelic ratio was low), NPM1 mutation was classified as part of “myeloid.gene.R”–a poor-prognosis cluster for AML–according to the Pan-Myeloid Axis. We, however, were not alone in finding this inconsistency regarding NPM1; previous researchers have published clinical outcome analyses that contradicted 2017 ELN with respect to the role of NPM1 mutation in prognosis (83–85). Similarly, CEBPA biallelic mutation was designated as favorable in 2017 ELN, but CEBPA mutation was classified as “myeloid.gene.R” in our study. The “2017 ELN enhanced” scheme reclassified some of the 2017 ELN-designated “favorable” patients in THAMP as intermediate-risk (“group 2”) because of their high total mutation load (>2) in “R + MR”, i.e., they had concomitant mutations in the other “high-risk” genes. It has been reported that not all CEBPA mutations carry favorable prognosis for AML (42); moreover, concomitant mutation in WT1 (classified as “myeloid.gene.R”) has been reported to predict poor prognosis in AML even when CEBPA biallelic mutations were present (86).

For MDS, the THAMP dataset provided evidence that the genes situated at the left end of the Pan-Myeloid Axis added independent prognostic value in adult MDS-EB beyond IPSS-R scoring, consistent with the previously reported adverse impacts of mutations in ASXL1, BCOR, BCORL1, CBL, CUX1, EP300, ETV6, EZH2, JAK2, NF1, PRPF8, PTPN11, SETBP1, SF3B1, SMC3, SRSF2, STAG2, and U2AF1 (all classified as “myeloid.gene.L” or “myeloid.gene.ML”; refs. 13, 29, 87–95).

Taken together, our pan-myeloid analysis provided comparative genomics evidence that primary myeloid neoplasms indeed form a continuum. In addition, we showed that variation of treatment response in individual patients is influenced by how the diseases are organized along the phenogenetically-defined Pan-Myeloid Axis. With the ever growing trend of redefining hematologic neoplasm entities in molecular terms (21, 96), diagnostic boundaries between the diseases and decision trees for treatment planning will continue to evolve and change shape in light of new genomics evidence. The ultimate test for the concept of “myeloid neoplasm continuum” would be systematic testing of successively graded and measured customization of treatment regimens along the Pan-Myeloid Axis.

## Supplementary Material

Figures S1-S6, Tables S8-S10 Figure S1. The most prevalent gene mutations and chromosomal abnormalities for AML in THAMP. Figure S2. The most prevalent gene mutations and chromosomal abnormalities for MDS in THAMP. Figure S3. The most prevalent gene mutations and chromosomal abnormalities for MDS/MPN in THAMP. Figure S4. The most prevalent gene mutations and chromosomal abnormalities for MPN in THAMP. Figure S5. Ranking order of genes along the Pan-Myeloid Axis. Figure S6. Ranking order of clinical features along the Pan-Myeloid Axis. Table S8. Clinical features in THAMP. Table S9. ELN risk stratification of AML patients in THAMP. Table S10. Classification and risk stratification of MDS patients in THAMP.

Table S1 Table S1. Clinicopathologic characteristics of the 730 myeloid neoplasm patients in THAMP.

Table S2 Table S2. Clinicopathologic characteristics of the 462 lymphoid neoplasm patients in THAMP.

Table S3 Table S3. 171 sequenced genes in THAMP.

Table S4 Table S4. Experimental validation of next-generation sequencing variant calls by Sanger sequencing.

Table S5 Table S5. THAMP-Illumina-171 and the 27 benchmark gene panels.

Table S6 Table S6. Gene mutation rates in THAMP and comparisons with AACR GENIE 10.0-public.

Table S7 Table S7. Prevalence of chromosomal abnormalities in THAMP.
