# Supplementary material for: A Phenogenetic Axis that Modulates Clinical Manifestation and Predicts Treatment Outcome in Primary Myeloid Neoplasms
Source: Cancer Res Commun. 2022 Apr 26;2(4):258–76. doi: 10.1158/2767-9764.CRC-21-0194 (PMC9981215; doi:10.1158/2767-9764.CRC-21-0194)
Supplement: Figures S1-S6, Tables S8-S10 — Figure S1. The most prevalent gene mutations and chromosomal abnormalities for AML in THAMP. Figure S2. The most prevalent gene mutations and chromosomal abnormalities for MDS in THAMP. Figure S3. The most prevalent gene mutations and chromosomal abnormalities for MDS/MPN in THAMP. Figure S4. The most prevalent gene mutations and chromosomal abnormalities for MPN in THAMP. Figure S5. Ranking order of genes along the Pan-Myeloid Axis. Figure S6. Ranking order of clinical features along the Pan-Myeloid Axis. Table S8. Clinical features in THAMP. Table S9. ELN risk stratification of AML patients in THAMP. Table S10. Classification and risk stratification of MDS patients in THAMP. [file crc-21-0194-s01.pdf]

Supplementary Data for

A phenogenetic axis that modulates clinical manifestation and predicts treatment outcome in primary myeloid neoplasms

Qiujin Shen, Yahui Feng, Xiaowen Gong, Yujiao Jia, Qingyan Gao, Xiaokang Jiao, Saibing Qi, Xueou Liu, Hui Wei, Bingqing Huang, Ningning Zhao, Xiaoqiang Song, Yueshen Ma, Shihao Liang, Donglei Zhang, Li Qin, Ying Wang, Shiqiang Qu, Yao Zou, Yumei Chen, Ye Guo, Shuhua Yi, Gang An, Zengtao Jiao, Song Zhang, Linfeng Li, Jun Yan, Huijun Wang, Zhen Song, Yingchang Mi, Lugui Qiu, Xiaofan Zhu, Jianxiang Wang*, Zhijian Xiao*, Junren Chen*

*Corresponding authors. Email: wangjx@ihcams.ac.cn; zjxiao@hotmail.com; chenjunren@ihcams.ac.cn

This PDF file includes:

Figs. S1 to S6
Tables S8 to S10

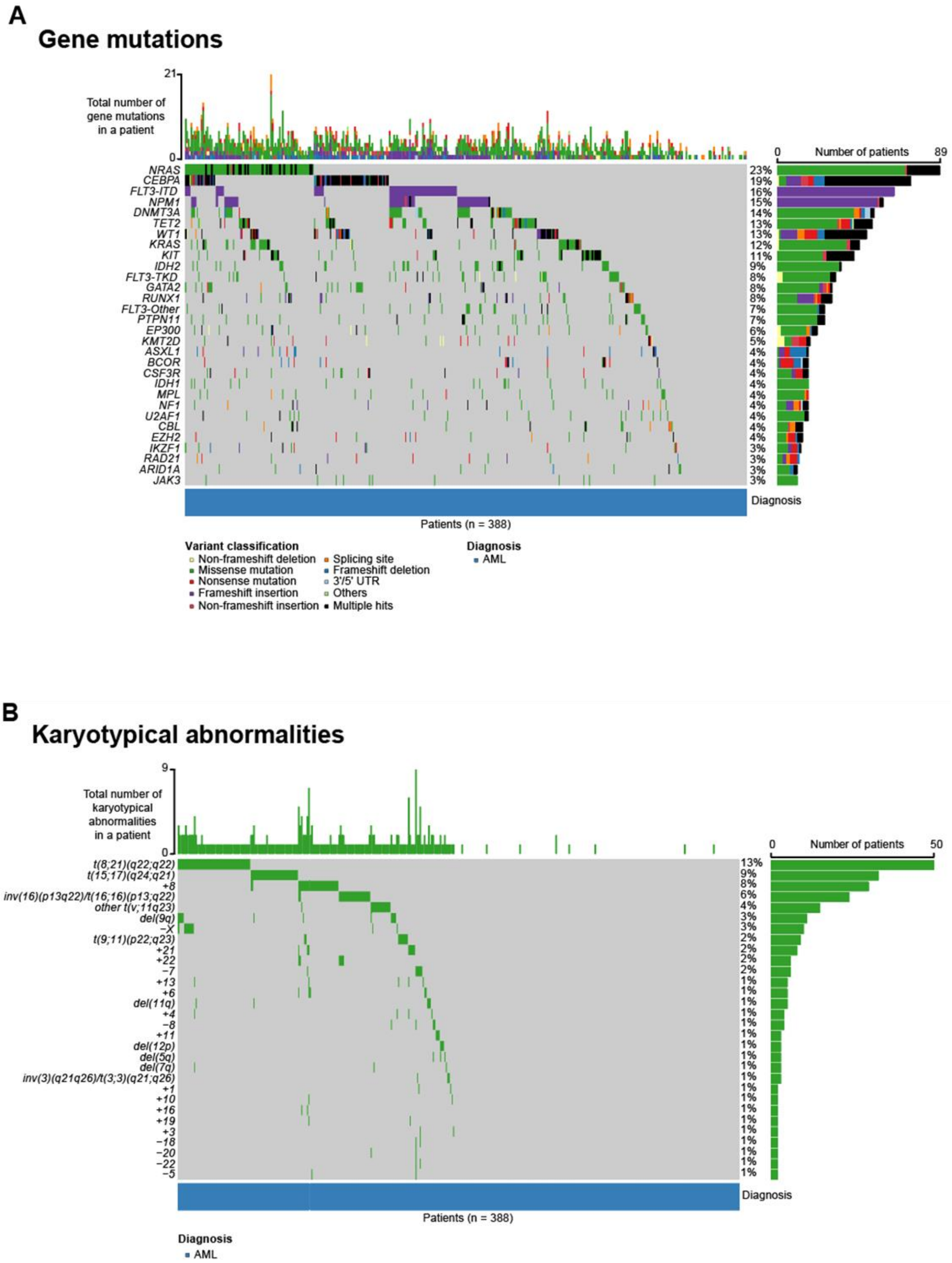

Fig. S1. The most prevalent gene mutations and chromosomal abnormalities for AML in THAMP.

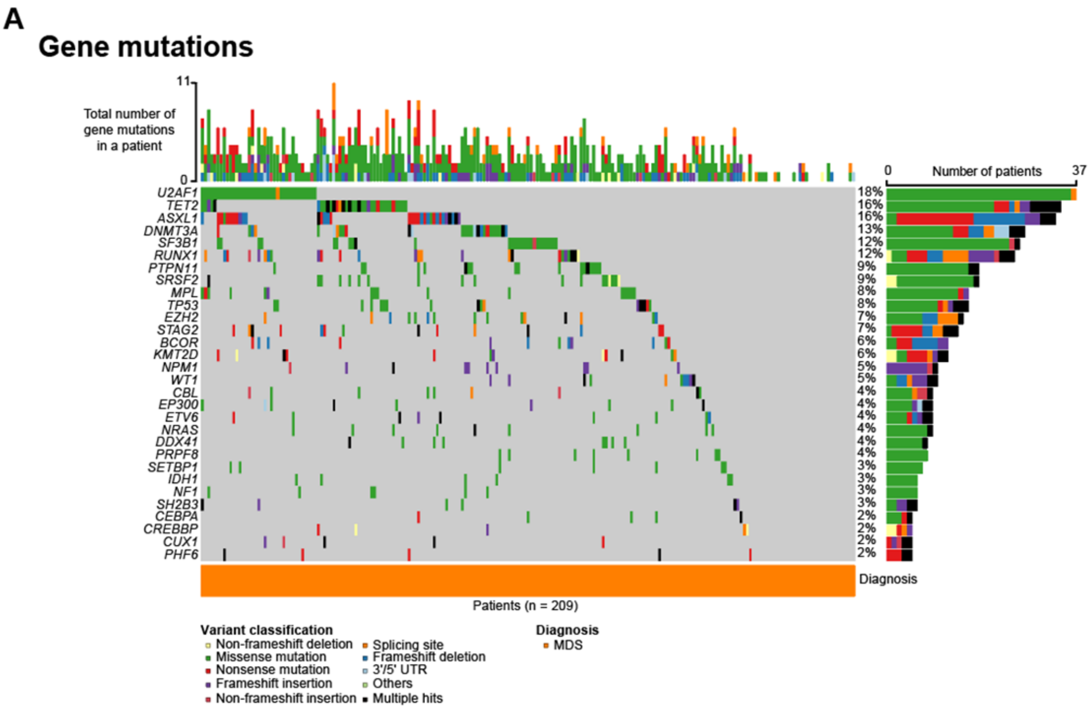

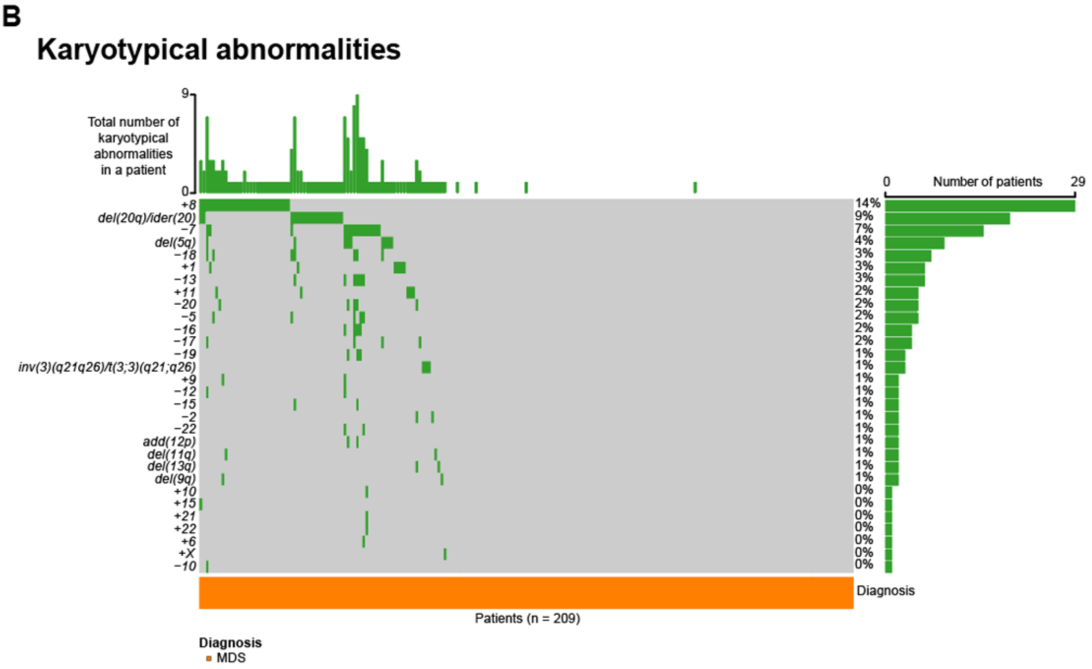

Fig. S2. The most prevalent gene mutations and chromosomal abnormalities for MDS in THAMP.

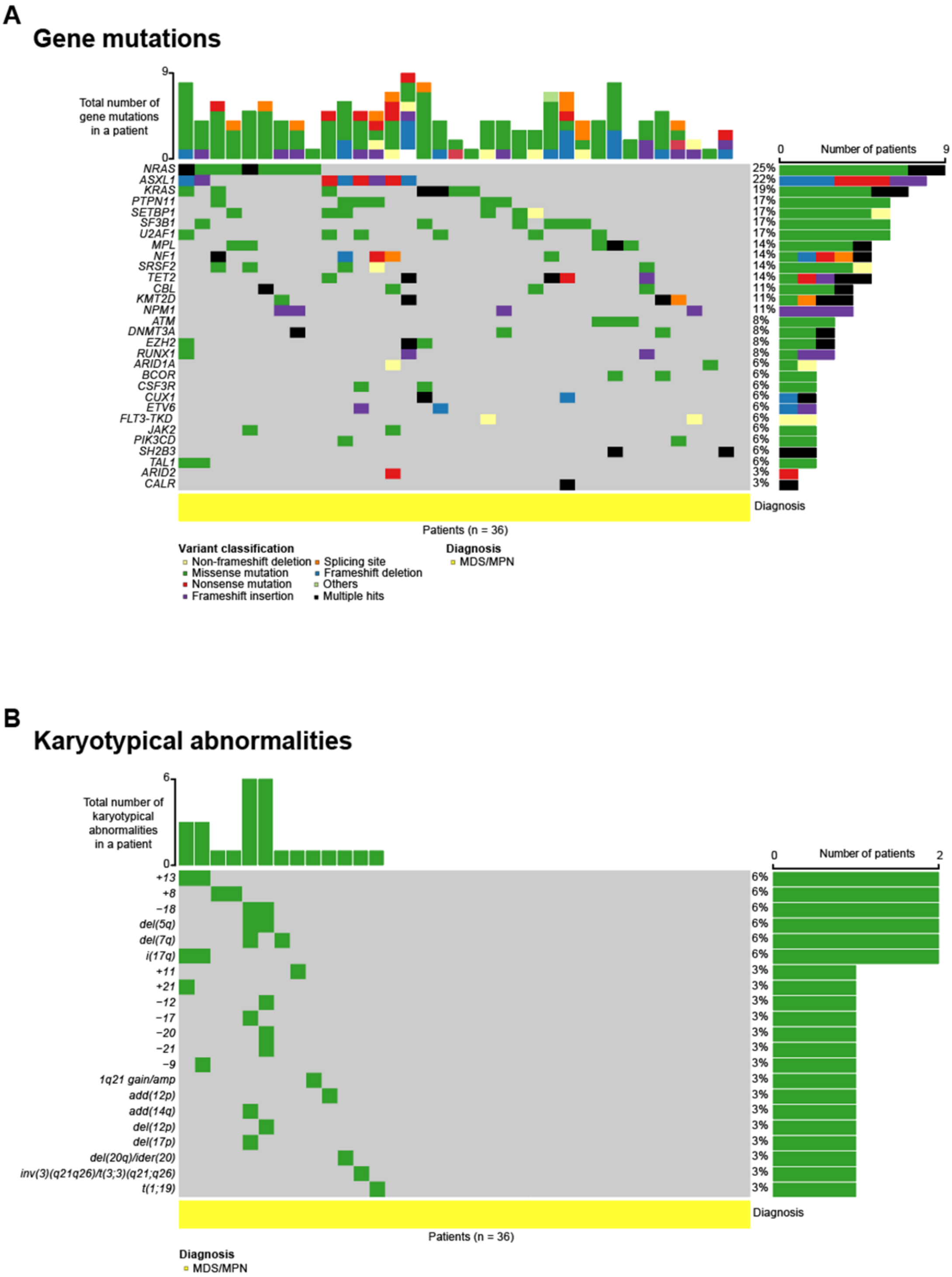

Fig. S3. The most prevalent gene mutations and chromosomal abnormalities for MDS/MPN in THAMP.

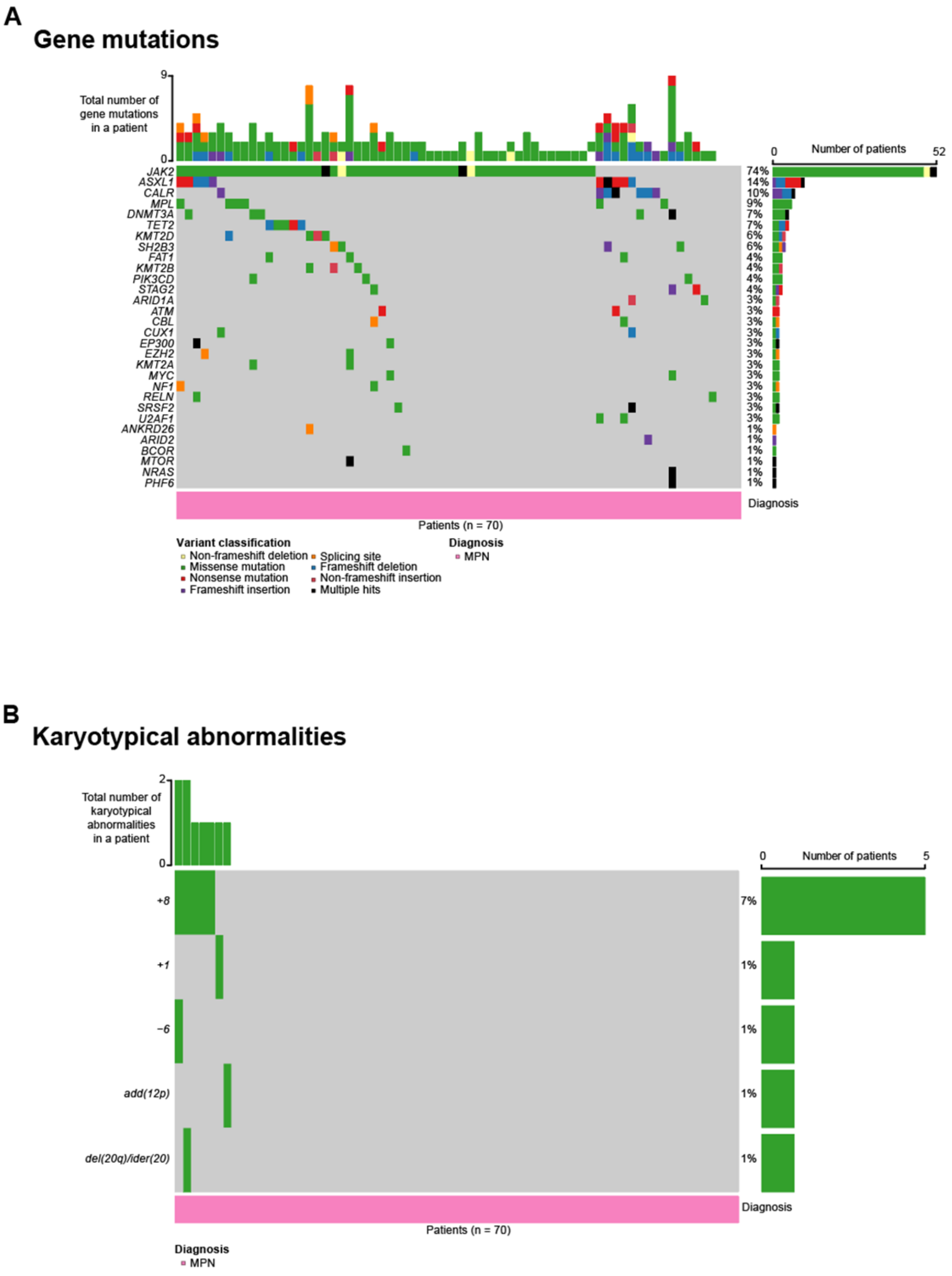

Fig. S4. The most prevalent gene mutations and chromosomal abnormalities for MPN in THAMP.

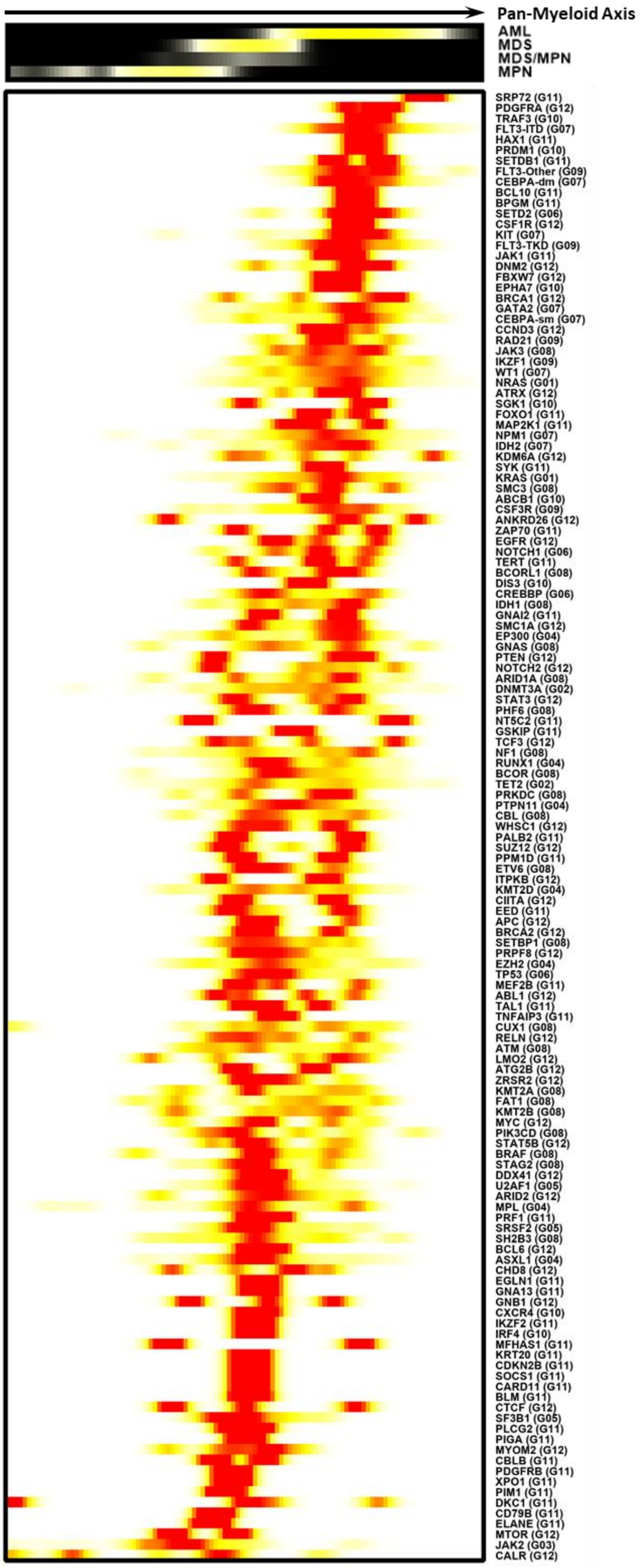

Fig. S5. Ranking order of genes along the Pan-Myeloid Axis.

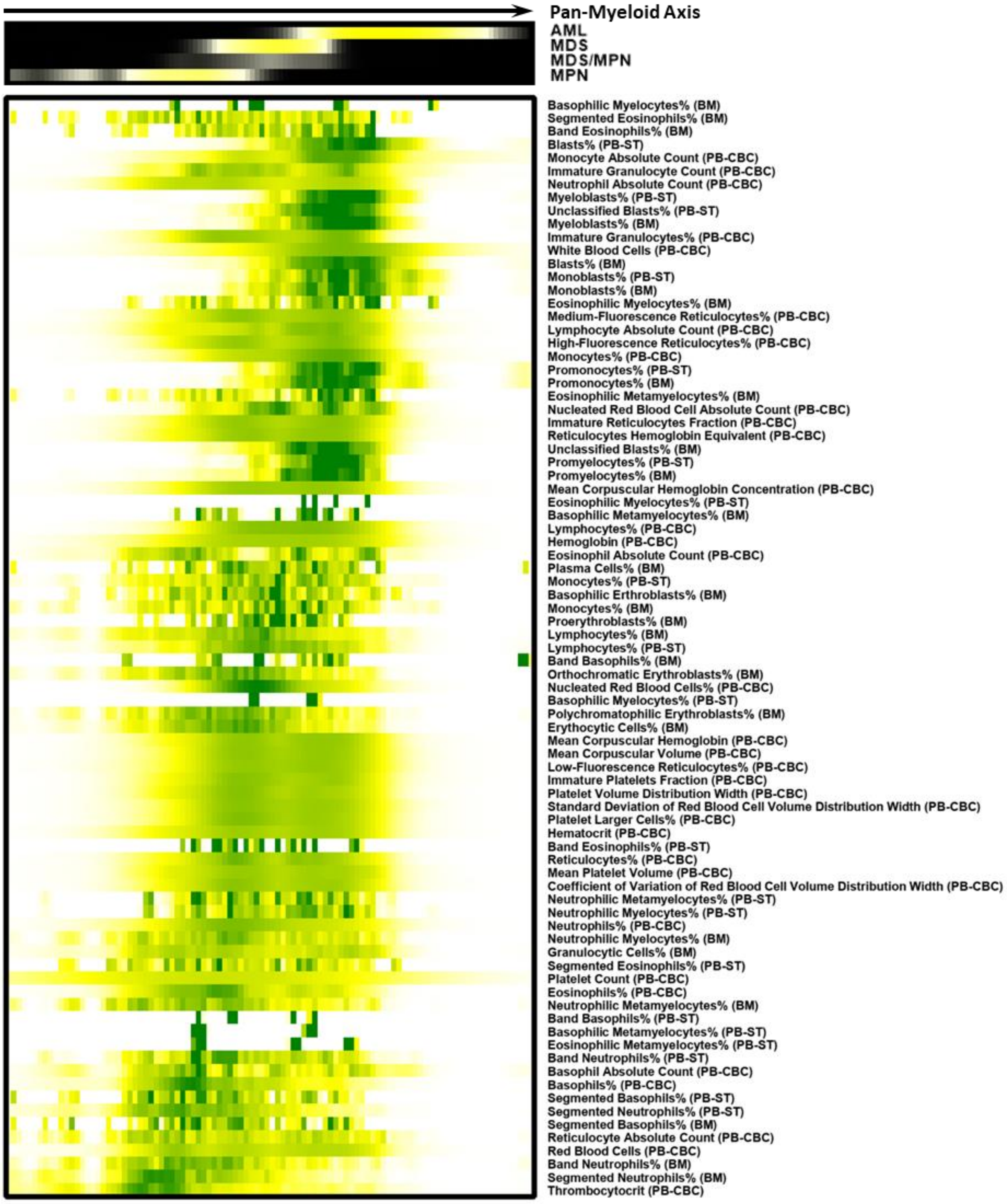

Fig. S6. Ranking order of clinical features along the Pan-Myeloid Axis.

Table S8. Clinical features in THAMP.

**Peripheral blood - complete blood count (PB-CBC)**		
Basophil Absolute Count, BASO#	Low-Fluorescence Reticulocytes%, LFR%	Nucleated Red Blood Cell Absolute Count, NRBC#
Basophils%, BASO%	Lymphocyte Absolute Count, LYMPH#	Nucleated Red Blood Cells%, NRBC%
Eosinophil Absolute Count, EO#	Lymphocytes%, LYMPH%	Platelet Count, PLT
Eosinophils%, EO%	Mean Corpuscular Hemoglobin, MCH	Platelet Larger Cells%, P-LCR%
Hematocrit, HCT	Mean Corpuscular Hemoglobin Concentration, MCHC	Platelet Volume Distribution Width, PDW
Hemoglobin, HGB	Mean Corpuscular Volume, MCV	Red Blood Cells, RBC
High-Fluorescence Reticulocytes%, HFR%	Mean Platelet Volume, MPV	Reticulocyte Absolute Count, RET#
Immature Granulocyte Count, IG#	Medium-Fluorescence Reticulocytes%, MFR%	Reticulocytes Hemoglobin Equivalent, RET-He
Immature Granulocytes%, IG%	Monocyte Absolute Count, MONO#	Reticulocytes%, RET%
Immature Platelets Fraction, IPF	Monocytes%, MONO%	Thrombocytocrit, PCT
Immature Reticulocytes Fraction, IRF	Neutrophil Absolute Count, NEUT#	Neutrophils%, NEUT%
White Blood Cells, WBC		
Coefficient of Variation of Red Blood Cell Volume Distribution Width, RDW-CV		
Standard Deviation of Red Blood Cell Volume Distribution Width, RDW-SD		
**Peripheral blood examination by pathologists** (100 cells)		
Abnormal Plasma Cells% (PB-ST)	Eosinophilic Myelocytes% (PB-ST)	Plasma Cells% (PB-ST)
Band Basophils% (PB-ST)	Lymphoblasts% (PB-ST)	Prolymphocytes% (PB-ST)
Band Eosinophils% (PB-ST)	Lymphocytes% (PB-ST)	Promonocytes% (PB-ST)
Band Neutrophils% (PB-ST)	Monoblasts% (PB-ST)	Promyelocytes% (PB-ST)
Basophilic Metamyelocytes% (PB-ST)	Monocytes% (PB-ST)	Segmented Basophils% (PB-ST)
Basophilic Myelocytes% (PB-ST)	Myeloblasts% (PB-ST)	Segmented Eosinophils% (PB-ST)
Blasts% (BM)	Neutrophilic Metamyelocytes% (PB-ST)	Segmented Neutrophils% (PB-ST)

Blasts% (PB-ST)	Neutrophilic Myelocytes% (PB-ST)	Unclassified Blasts% (PB-ST)
Eosinophilic Metamyelocytes% (PB-ST)	Plasma Blasts% (PB-ST)	
**Bone marrow examination by pathologists** (200 cells)		
Abnormal Plasma Cells% (BM)	Granulocytic Cells% (BM)	Plasma Cells% (BM)
Band Basophils% (BM)	Lymphoblasts% (BM)	Polychromatophilic Erythroblasts% (BM)
Band Eosinophils% (BM)	Lymphocytes% (BM)	Prolymphocytes% (BM)
Band Neutrophils% (BM)	Monoblasts% (BM)	Promonocytes% (BM)
Basophilic Erthroblasts% (BM)	Monocytes% (BM)	Promyelocytes% (BM)
Basophilic Metamyelocytes% (BM)	Myeloblasts% (BM)	Proerythroblasts% (BM)
Basophilic Myelocytes% (BM)	Neutrophilic Metamyelocytes% (BM)	Segmented Basophils% (BM)
Eosinophilic Metamyelocytes% (BM)	Neutrophilic Myelocytes% (BM)	Segmented Eosinophils% (BM)
Eosinophilic Myelocytes% (BM)	Orthochromatic Erythroblasts% (BM)	Segmented Neutrophils% (BM)
Erythocytic Cells% (BM)	Plasma Blasts% (BM)	Unclassified Blasts% (BM)

PB-CBC, peripheral blood - complete blood count; PB-ST, peripheral blood - smear test.

Table S9. ELN risk stratification of AML patients in THAMP.

	AML*		
	**Total (n)**	**Children (n (%))**	**Adults (n (%))**
	349	59 (16.9)	290 (83.1)
**2017 ELN risk score**			
**Favorable**	168	25 (14.9)	143 (85.1)
**Intermediate**	97	21 (21.6)	76 (78.4)
**Adverse**	84	13 (15.5)	71 (84.5)
**AML with myelodysplasia-related changes**	28	6 (21.4)	22 (78.6)
**Cytogenetic abnormality**	27	6 (22.2)	21 (77.8)
**Multilineage dysplasia**	3	1 (33.3)	2 (66.7)
**Treatment**			
**Standard induction therapy**	276	55 (19.9)	221 (80.1)
**Use HMA**	39	2 (5.1)	37 (94.9)
**Use Venetoclax**	5	0 (0.0)	5 (100.0)
**Use Venetoclax and HMA**	4	0 (0.0)	4 (100.0)
**Others**	25	2 (8.0)	23 (92.0)
**CR status available**			
**Yes**	302	55 (18.2)	247 (81.8)
**No**	47	4 (8.5)	43 (91.5)

* AML-M3 cases were excluded from ELN classification.

2017 ELN, 2017 European LeukemiaNet; HMA, hypomethylating agent; CR, complete response.

Table S10. Classification and risk stratification of MDS patients in THAMP.

	MDS		
	**Total (n)**	**Children (n (%))**	**Adults (n (%))**
	209	10 (4.8)	199 (95.2)
**2016 WHO category**			
**MDS-5q–**	2	0 (0.0)	2 (100.0)
**MDS-SLD**	10	0 (0.0)	10 (100.0)
**MDS-MLD**	61	1 (1.6)	60 (98.4)
**MDS-RS**	19	0 (0.0)	19 (100.0)
**MDS-EB**	101	8 (7.9)	93 (92.1)
**MDS-U**	15	0 (0.0)	15 (100.0)
**MDS-RCC**	1	1 (100.0)	0 (0.0)
**IPSS-R**			
**Very good**	4	0 (0.0)	4 (100.0)
**Good**	41	0 (0.0)	41 (100.0)
**Intermediate**	64	0 (0.0)	64 (100.0)
**Poor**	52	3 (5.8)	49 (94.2)
**Very poor**	48	7 (14.6)	41 (85.4)

MDS-5q–, MDS with isolated deletion of long arm of chromosome 5; MDS-SLD, MDS with single lineage dysplasia; MDS-MLD, MDS with multilineage dysplasia; MDS-RS, MDS with ring sideroblasts; MDS-EB, MDS with excess blasts; MDS-U, unclassifiable MDS; MDS-RCC, MDS with refractory cytopenia of childhood; IPSS-R, Revised International Prognostic Scoring System.
